# A Unique Cellular and Molecular Microenvironment Is Present in Tertiary Lymphoid Organs of Patients with Spontaneous Prostate Cancer Regression

**DOI:** 10.3389/fimmu.2017.00563

**Published:** 2017-05-17

**Authors:** María de la Luz García-Hernández, Norma Ofelia Uribe-Uribe, Ricardo Espinosa-González, W. Martin Kast, Shabaana A. Khader, Javier Rangel-Moreno

**Affiliations:** ^1^Department of Medicine, University of Rochester, Rochester, NY, USA; ^2^Department of Anatomy and Anatomical Pathology, Instituto Nacional de Ciencias Medicas y Nutricion Salvador Zubiran, Mexico City, Mexico; ^3^Department of Molecular Microbiology and Immunology, Norris Comprehensive Cancer Center, University of Southern California, Los Angeles, CA, USA; ^4^Department of Urology, Norris Comprehensive Cancer Center, University of Southern California, Los Angeles, CA, USA; ^5^Department of Obstetrics and Gynecology, University of Southern California, Los Angeles, CA, USA; ^6^Department of Molecular Microbiology, Washington University in Saint Louis, St. Louis, MO, USA

**Keywords:** tertiary lymphoid organs, high endothelial venules, homeostatic chemokines, evanescent prostate carcinoma, cyclooxygenase 2, prostatic intraepithelial neoplasia, follicular dendritic cells, peripheral node addressin

## Abstract

**Objective:**

Multiple solid cancers contain tertiary lymphoid organs (TLO). However, it is unclear whether they promote tumor rejection, facilitate tumor evasion, or simply whether they are a byproduct of chronic inflammation. We hypothesize that although chronic inflammation induces TLO formation, the tumor milieu can modulate TLO organization and functions in prostate cancer. Therefore, our study seeks to elucidate the cellular and molecular signatures in unique prostatectomy specimens from evanescent carcinoma patients to identify markers of cancer regression, which could be harnessed to modulate local immunosuppression or potentially enhance TLO function.

**Methods:**

We used multicolor immunofluorescence to stain prostate tissues, collected at different stages of cancer progression (prostatic intraepithelial neoplasia, intermediate and advanced cancer) or from patients with evanescent prostate carcinoma. Tissues were stained with antibodies specific for pro-inflammatory molecules (cyclooxygenase 2, CXCL10, IL17), tumor-infiltrating immune cells (mature DC-LAMP^+^ dendritic cells, CD3^+^ T cells, CD3^+^Foxp3^+^ regulatory T cells (Treg), T bet^+^ Th1 cells, granzyme B^+^ cytotoxic cells), and stromal cell populations (lymphatic vessels, tumor neovessels, high endothelial venules (HEV), stromal cells), which promote prostate tumor growth or are critical components of tumor-associated TLO.

**Results:**

Generally, inflammatory cells are located at the margins of tumors. Unexpectedly, we found TLO within prostate tumors from patients at different stages of cancer and in unique samples from patients with spontaneous cancer remission. In evanescent prostate carcinomas, accumulation of Treg was compromised, while Tbet^+^ T cells and CD8 T cells were abundant in tumor-associated TLO. In addition, we found a global decrease in tumor neovascularization and the coverage by cells positive for cyclooxygenase 2 (COX2). Finally, consistent with tumor regression, prostate stem cell antigen was considerably reduced in TLO and tumor areas from evanescent carcinoma patients.

**Conclusion:**

Collectively, our results suggest that COX2 and Treg are attractive therapeutic targets that can be harnessed to enhance TLO-driven tumor immunity against prostate cancer. Specially, the presence of HEV and lymphatics indicate that TLO can be used as a platform for delivery of cell-based and/or COX2 blocking therapies to improve control of tumor growth in prostate cancer.

## Introduction

Prostate cancer is the second malignancy diagnosed among men around the world (1), and it is the second cause of cancer death in United States (2). Fortunately, the mortality rate in patients with mild disease has decreased in the last 30 years after the introduction of prostate-specific antigen (PSA) screening. However, The American Cancer Society reported 180,980 new cases of prostate cancer and 26,120 deaths in 2016. This indicates that still better treatments are needed to improve survival for prostate cancer patients. For many years, Gleason score has been the classical tool to estimate progression and aggressiveness in prostate cancer and has helped to guide personalized therapy. Although it is based on detecting morphological changes in the prostate gland epithelium, recent findings in the field of solid cancer suggests that most attention should be invested on searching for organized collections of tumor-infiltrating immune cells (T cells, B cells, dendritic cells), which are associated with improved survival prognosis and can reveal therapeutic targets for the clinical benefit of prostate cancer patients.

These organized collections of lymphocytes are known as tertiary lymphoid structures or tertiary lymphoid organs (TLO) and arise in peripheral tissues as a consequence of persistent inflammatory or antigenic stimulation (3–5). In seminal studies performed by Nancy Ruddle’s group, it was proposed that chronic inflammation was the driving force behind TLO formation (6, 7). Later, TLO were found in the liver and stomach of mice and in humans infected with *Helicobacter pylori*. In this particular setting, the stability of TLO was disrupted by reduction of the bacterial load after administration of antibiotics (8, 9). These findings suggest that antigens and recognition of pathogen- or damage-associated molecular patterns by immune cells are actively participating in TLO formation/organization, and that reduction in the local antigenic burden compromises TLO integrity. Finally, although it is proposed that LT and HC chemokines are crucial for TLO formation and organization, several studies have recently revealed the contribution of additional inflammatory cytokines in TLO formation (10–14). In general, it is proposed that TLO have flexible programs, which are associated with protection (infectious disease, cancer) or that contribute to local pathology in inflammatory and autoimmune diseases (chronic obstructive pulmonary disease, rheumatoid arthritis, multiple sclerosis, Sjogren syndrome) (15–17). However, TLO functions can be both shaped by the local environment and influenced by temporal changes in disease- and tissue-specific signals. Interestingly, it is proposed that the immunosuppressive environment in tumors should interfere with TLO formation (18, 19). Thus, a better characterization of the local milieu will likely contribute to having a better understanding of the cellular and molecular factors that stabilize or disrupt TLO formation and organization. A basic understanding about the contexture of the complex cellular and molecular microenvironments surrounding TLO will provide the key to design therapeutic approaches that modulate TLO formation, organization, and stability for the benefit of patients afflicted by inflammatory, autoimmune, infectious, and malignant diseases.

Tertiary lymphoid organs have been detected in solid cancers affecting a variety of tissues (breast, skin, lung, pancreas) (19–21). The assumption is that TLO are facilitating the induction of local immunity that participates in tumor clearance. Interestingly, although Di Carlo et al. reported presence of a TLO in the prostate of healthy individuals (22), there is scarce information about the presence of TLO at different stages of prostate cancer (23). In the majority of solid tumors, infiltrating immune cells and TLO are preferentially located in the periphery of malignant tissue (24, 25). Unexpectedly, we detected tumor-associated TLO in the prostate of patients at different stages of disease, ranging from prostatic intraepithelial neoplasia (PIN) to late stages of prostate cancer. Importantly, they were even present in the prostate of a unique group of cancer patients that experienced spontaneous remission. This initial observation showed us that despite that TLO are present at different stages of cancer progression; their functions may be influenced by the spatiotemporal dynamic changes in the tumor environment. Thus, the central goal of this study was focused on defining the cellular and molecular changes in the tumor environment around TLO, which might be associated with tumor progression or regression and that are likely modulating TLO functions.

## Materials and Methods

### Prostate Tissue and Patient Information

Prostate specimens were collected with written consent of patients and after approval by the Ethical Committee of the National Institute of Medical Sciences and Nutrition “Salvador Zubiran.” A total of 27 prostate specimens (14 biopsies and 13 prostatectomy specimens) from 17 patients were included in our retrospective study. Interestingly, four patients who were initially diagnosed with adenocarcinoma (biopsy) had no evidence of tumor growth after subsequent collection of prostatectomy specimens—a histopathological finding commonly recognized by urologists as evanescent prostate carcinoma (26). Importantly, none of the patients were treated with any medication before prostatectomy, ruling out the possibility that cancer therapy caused tumor regression. We divided our patients in two cohorts: evanescent (*n* = 4) and non-evanescent prostate carcinoma (*n* = 13). Additionally, patients in the non-evanescent cohort were classified according to prostate cancer aggressiveness (Gleason grading system) (27) into intermediate and advanced prostate carcinoma groups by two certified urologists.

### Antibodies

#### Primary Antibodies

The primary antibodies were as follows: goat anti-human CD105 (AF1097, R&D Systems), rabbit anti cyclooxygenase 2 (GTX15191, GeneTex), mouse anti-human CD68 (clone PG-M1, GeneTex), goat anti-CD3 epsilon (clone M-20, Santa Cruz Biotechnology), rabbit anti-T bet (H-210, Santa Cruz biotechnology), rat anti-human Foxp3 (PCH101, eBioscience), goat-anti proliferating cell nuclear antigen (PCNA) (clone C-20, Santa Cruz Biotechnology), mouse anti-human Ki-67 (clone MIB-1, Dakocytomation), rabbit anti-human CD8 (clone SP16, Thermo Fisher Scientific), mouse anti-human CD20 (clone L-26, Abcam), rabbit anti-human PD-L1 (Invitrogen, PA5-28115), rabbit anti-IL17 (H-132, Santa Cruz Biotechnology), mouse anti-human CD21 (clone 2G9, Thermo Fisher Scientific), rat anti-peripheral node addressin (clone MECA-79, BD Pharmigen), rabbit anti-human CD138 (RB-9422-P1, Thermo Fisher Scientific), mouse anti-podoplanin (clone D2-40, GTX31231, GeneTex), rabbit anti-granzyme B (clone EPR8260, Abcam), rat anti-human DC-LAMP (clone 1010E1.01, Novus Biologicals), rabbit anti-human prostate stem cell antigen (PSCA) (GTX15168, GeneTex), rabbit anti-human CXCL10 (GTX31176, GeneTex), biotin-mouse anti-smooth muscle actin (clone 1A4, Thermo Fisher Scientific), and mouse anti-human plasma cell (clone LIV3G11, Thermo Fisher Scientific).

#### Secondary Antibodies and Streptavidin

The secondary antibodies and streptavidin were as follows: Alexa Fluor 568-donkey anti-goat Ig G (H + L) cross adsorbed (A-11057, Thermo Fisher Scientific), F(ab)^2^ FITC-donkey anti-rabbit (711-096-152, Jackson ImmunoResearch Laboratories), F(ab)^2^ biotin-donkey anti mouse (715-066-150, Jackson ImmunoResearch Laboratories), F(ab)^2^ biotin-donkey anti-rat Ig G (712-066-150, Jackson ImmunoResearch Laboratories), Cy3-goat anti-rat Ig M (112-166-075), and Cy5-streptavidin (405209, Biolegend).

### Immunofluorescent Detection of Cell Infiltrates in Paraffin Prostate Sections

The 5-μm paraffin tissue sections were first incubated at 60°C for at least 1 h to melt paraffin, followed by quick transfer into xylenes. After removing paraffin, slides were hydrated by sequential immersion into absolute alcohol, 95% alcohol, 75% alcohol, and finally thoroughly washed with deionized water. Antigens were unmasked by boiling slides for 30 min in antigen retrieval solution (S1699, Dako laboratories). To prevent non-specific binding, sections were incubated for 30 min with 5% normal donkey serum (017-000-121, Jackson ImmunoResearch Laboratories). Next, primary antibodies were added to prostate sections and incubated overnight at room temperature. To reveal primary antibodies, slides were incubated at room temperature for 2 h with fluorescent secondary antibodies. Sections were incubated with Cy5-conjugated streptavidin (405209, eBioscience) for one extra hour at room temperature to detect biotinylated antibodies. Finally, sections were washed with PBS and mounted with Vectashield mounting medium with DAPI (H-1200, Vector Laboratories). Representative pictures were acquired with a Zeiss Axioplan 2 microscope and recorded with a Hamamatsu camera after performing morphometric analysis.

### Morphometric Analysis of Lymphoid and B Cell Follicles

Lymphoid follicles (LF) were defined as organized and compact collections of lymphocytes that were readily detected in H&E sections from prostatectomy specimens. For morphometric analysis of LF, all organized lymphocytic structures in individual prostatectomy specimens were enumerated and outlined with automated tools of the Zeiss Axioplan software to calculate the average number and size of LF. LF dimensions are expressed in square microns.

### Area Covered by CD105^+^ Vessels, COX2^+^, PSCA^+^, and CXCL10^+^ Cells, in TLO-Associated Microenvironments and Tumor Areas Lacking TLO

Nine 200× pictures were automatically stitched with the mosaic feature of the Zeiss Axioplan 2 microscope software (mosaic pictures: 3 × 3 200× pictures). TLO were placed in the center of the mosaics to have a global view of surrounding tumor microenvironment. We acquired JPGE mosaic pictures of all TLO areas or five to eight mosaic pictures from tumor areas without TLO in each prostatectomy section, and measured the area occupied by CD105^+^ vessels, COX2^+^, PSCA^+^, and CXCL10^+^ cells in a mosaic picture with NIH ImageJ software. Measurements using ImageJ software were blindly performed by two independent evaluators and using the same parameters for all the pictures. The percentage of area covered by CD105^+^ vessels, COX2^+^, PSCA^+^, and CXCL10^+^ cells in panoramic mosaics was calculated by dividing the area occupied by CD105^+^ vessels or positive cells (pixels^2^), by the total area of the panoramic mosaic (mosaic area = 1.043 μm^2^ = 10,034,185 pixels^2^).

### Quantitation of Granzyme B^+^ Cells, Regulatory T Cells (Treg), and DC LAMP^+^ Cells in Tumor-Associated TLO

DC LAMP, granzyme B, and CD8 T cells were counted in T cell areas of all TLO contained in individual prostate sections (200× random fields/section). In addition, the same populations were enumerated in five to eight random 200× fields per tissue section in areas with signs of epithelial cell transformation. We calculated the relative frequency of CD3^+^Foxp3^+^ (Treg/Treg + Tbet × 100) and CD3^+^Tbet^+^ (Tbet/Treg + Tbet × 100) in TLO. We also determined the ratio between Tbet^+^ T cells and Treg populations inside TLO to have an idea about the dynamic changes in those T cell populations during cancer progression/regression, and determined the correlation between the number of Treg and TLO in prostatectomy specimens from intermediate and advanced prostate cancer patients.

### Statistics

Comparisons between two groups were performed with two-tailed *T* test. Correlation was calculated with Pearson’s coefficient. Percentage of cancer-free patients after cancer diagnosis was estimated by Kaplan–Meier method, and significant differences among the groups were calculated by using long-rank (Mantel–Cox) test. Differences with a *p* value ≤0.05 were considered statistically significant.

## Results

### A Unique Cohort of Prostate Cancer Patients Experienced Spontaneous Disease Remission

**Figure 1:**
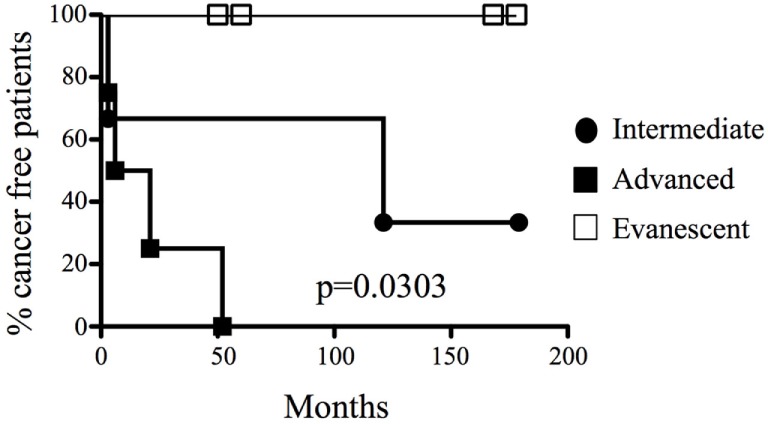
**A unique cohort of prostate cancer patients experienced spontaneous cancer remission**. Different groups of prostate cancer patients were classified according to their systemic levels of prostate antigen-specific antigen and histopathological features (biopsies or prostatectomy specimens) and were monitored for a maximal period of 179 months (approximately 15 years). 100% of patients were cancer free in low and evanescent carcinoma cohorts, compared to the rapid development of active malignant disease in patients with advanced prostate cancer (median for cancer development: 13.5 months), and the moderate cancer progression at intermediate stages of prostate cancer (median for cancer development: 121 months). Percentage of tumor-free patients was calculated by long rank test (Mantel–Cox). Differences in tumor development among the groups were statistically significant (*p* = 0.0303). *n* = 17 prostate cancer patients and 27 prostate specimens.

We collected 27 histological samples from 17 patients diagnosed with non-evanescent (intermediate and advanced grades) and evanescent prostate carcinoma. Patients with non-evanescent prostate carcinoma displayed clear histological signs of PIN (69%), considerable cancer aggressiveness (50% patients with a Gleason score of 8 and above), increased levels of PSA (83.5 ± 252.2), and showed clinical and pathological features of cancer progression (TNM stages: IIA to IV). By contrast, patients with evanescent carcinoma do not have any signs of prostate intraepithelial neoplasia (0%), had considerably lower PSA levels (12.2 ± 6.1), cancer was significantly less aggressive (6.0 ± 0.0), and did not have any evidence of clinical or pathological changes in the prostate (Table 1). We followed the patients for a maximal period of 179 months. As expected, we found that none of the patients diagnosed with advanced carcinoma were cancer free at 52 months post-diagnosis. By contrast, 33.3% of patients at intermediate stages of prostate cancer remained cancer free until the end of our retrospective study (179 months after cancer diagnosis). Interestingly, 100% of patients with evanescent prostate carcinoma were disease free at the conclusion of the study (Figure 1). Evanescent prostate carcinoma patients had evidence of prostate cancer in an initial biopsy but did not show any histological features of adenocarcinoma after collection of prostatectomy specimens for confirmatory diagnosis. Thus, we considered those prostatectomy specimens from patients with evanescent prostate cancer unique, because they could reveal therapeutic targets that can be harnessed to design novel prostate cancer therapies.

**Table 1:** **Demographic and clinical features of patients with prostatic carcinoma**.

	Evanescent carcinoma (*n* = 4)	Non-evanescent carcinoma (*n* = 13)
Age at diagnosis	66.3 ± 6.8	65.9 ± 5.6
Presence of prostatic intraepithelial neoplasia (yes/no)	0 (0%)/4 (100%)	9 (69%)/4 (31%)
Gleason sum (6/7/8/9/10)	4/0/0/0/0	2/5/2/3/1
Prostate-specific antigen at diagnosis	12.2 ± 6.1	83.5 ± 252.2
Extension of neoplasm in biopsy/prostatectomy (5)	1 ± 0	22.5 ± 24
Multicentricity (yes/no)	0 (0%)/4 (100%)	3 (23%)/10 (67%)
Perineural invasion (yes/no)	0 (0%)/4 (100%)	4 (31%)/9 (69%)
Necrotic tissue in tumor (yes/no)	0 (0%)/4 (100%)	4 (31%)/9 (69%)
Margins free of disease (yes/no)^a^	NA	4 (31%)/9 (69%)
Pathologic TNM stage (IIA/IIB/IIIIV)^a^	NA	1 (11%)/3 (33%)/4 (45%)/1 (11%)
Clinical TNM stage (I/IIA/IIB/III/IV)	10/0/0/0/0	1 (7.5%)/4 (31%)/1 (7.5%)/3 (23%)/4 (31%)

*^a^Information not available for patients who did not undergo a prostatectomy or whose prostatectomy did not contain tissue consistent with prostatic carcinoma*.

### Tumor-Associated LF Are Present in the Prostate during Cancer Progression and in Patients Experiencing Spontaneous Cancer Remission

**Figure 2:**
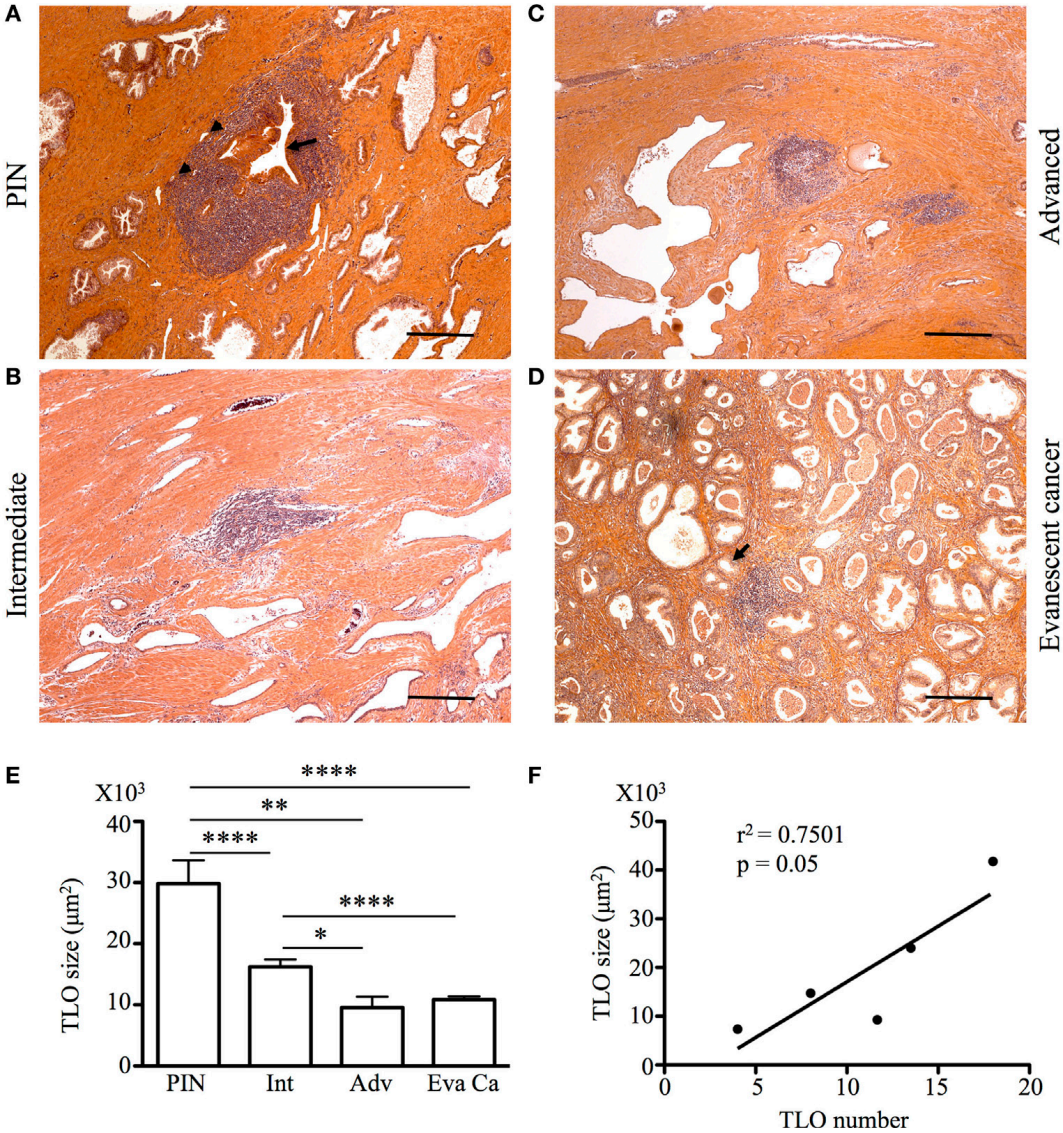
**Tumor-associated tertiary lymphoid organs (TLO) are present during prostate cancer progression and remission**. The 5-μm thick paraffin sections were stained with H&E, and all lymphoid follicles (LF) were enumerated and measured in a blinded manner. **(A–D)** After calculating the average size of LF, representative 200× magnification pictures from prostatectomy specimens of patients at different stages of cancer progression and patients with evanescent prostate carcinoma were taken with a Zeiss Axioplan microscope. Tumor-associated TLO were located in close proximity to epithelium (black arrows) and blood vessels (black arrowheads). Scale bar represents 100 μm. All LF in individual prostatectomy specimens were outlined with an automated tool of the Zeiss Axioplan microscope. **(E)** Average size of LF was calculated for prostatic intraepithelial neoplasia (PIN), intermediate (Int), advanced (Adv), and evanescent prostate carcinoma (Eva Ca). *n* = 28–97 measurements per group. **(F)** Significant correlation between number and size of TLO size during cancer progression is shown (Pearson, *r*^2^ = 0.7501, *p* = 0.05). Bar represent mean ± SEM. Statistically significant differences (*p ≤ 0.05, **p ≤ 0.005, ****p < 0.0001) were calculated by using two-tailed Student’s *t*-test with GraphPad Prism.

Tertiary lymphoid organs are induced in the context of chronic inflammation, autoimmunity, and cancer (24, 25) and are usually absent in healthy tissues. However, TLO have been previously described in the prostate of healthy individuals (22). Thus, considering the relevance of TLO in the positive prognosis of other solid malignancies (19), we examined the presence of organized collections of tumor-infiltrating lymphocytes in biopsies and prostatectomy specimens from patients with PIN, intermediate and advanced cancer, as well as in patients with evanescent carcinoma. Although we easily identified lymphocytic structures at all stages of prostate cancer (Figures 2A–C), and in prostatectomy specimens from patients with evanescent prostate carcinoma (Figure 2D), their sizes were very heterogeneous. Organized lymphocyte clusters were preferentially located inside tumors, in close proximity to glandular epithelium and blood vessels (Figures 2A,B). To define the average size of lymphocytic accumulations in prostatectomy specimens from PIN and prostate cancer patients, we outlined all TLO contained in individual sections with an automated tool of the Zeiss Axioplan software. TLO in PIN samples were significantly bigger (29,858.51 ± 23,608.22 μm^2^) than those in intermediate (16,218.62 ± 11,337.79 μm^2^, *p* < 0.0001), advanced (9,539.11 ± 6,608.31 μm^2^, *p* = 0.0027), and evanescent carcinoma (Eva Ca; 10,843.25 ± 5,274 μm^2^, *p* < 0.0001) (Figure 2E). Additionally, we found a directly proportional correlation between the number and the size of LF at intermediate and advanced stages of prostate carcinoma (*r*^2^ = 0.7501, *p* = 0.05) (Figure 2F). Our results are indicating that either a decline in the tumor antigen load or the immunosuppressive tumor environment is having a negative impact on the formation or stability of LF (19, 22, 24, 25).

### Tumor-Associated TLO in Prostate Cancer Contain Follicular Dendritic Cells (FDC), and Proliferating B Cells and CD8 T Cells

**Figure 3:**
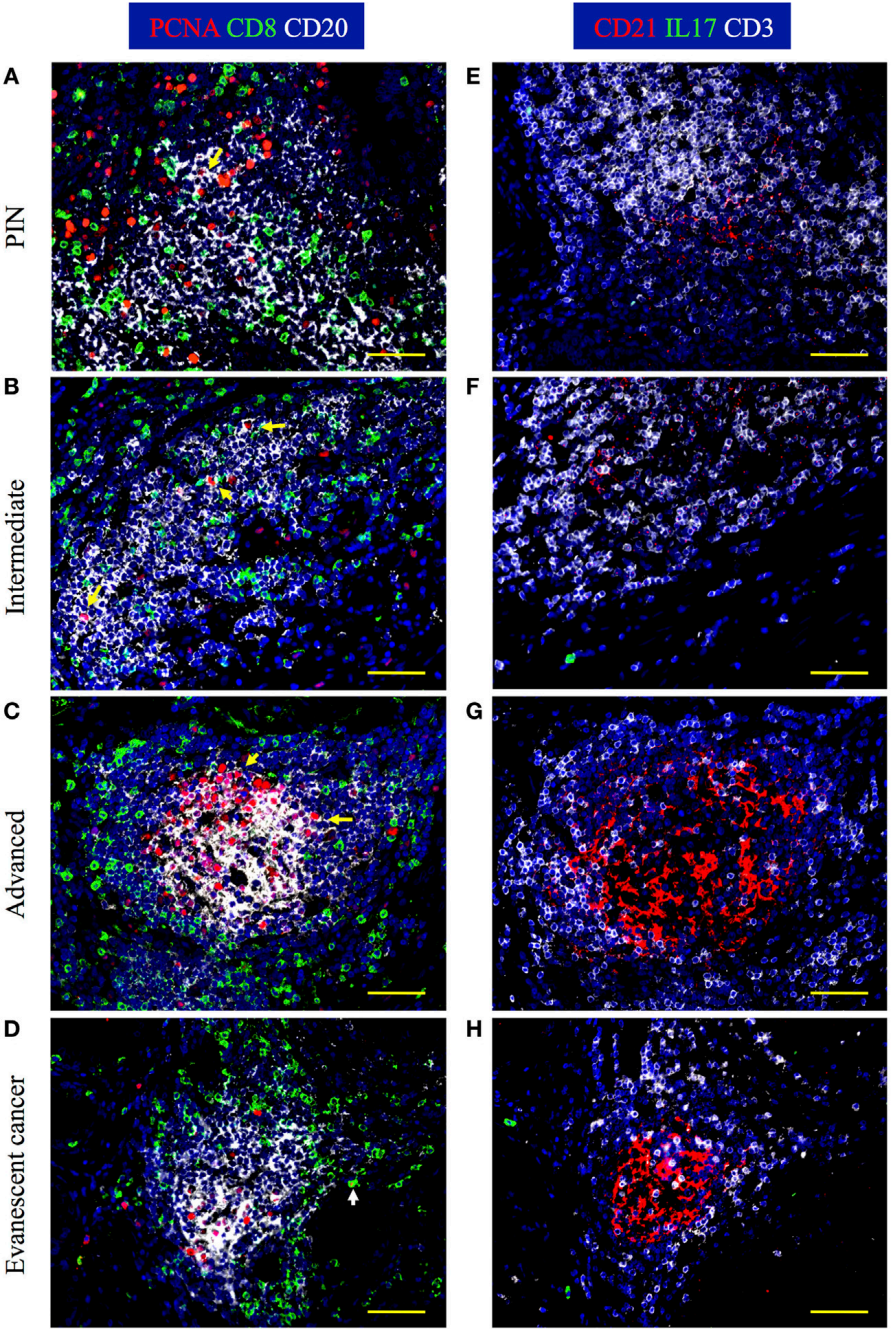
**IL17 production, B cell activation, and CD8 T cell accumulation are evident in prostate tumor-associated tertiary lymphoid organs (TLO)**. The 5-μm thick paraffin sections were stained with antibodies against proliferating cell nuclear antigen (PCNA, red), CD8 (green), and CD20 (white) to identify B cells and CD8 T cells in tumor-associated TLO. Serial sections were stained with antibodies against CD21 (red), IL17 (green), and CD3 (white). Representative 200× magnification pictures from the same TLO areas showed in H&E stains were taken with a Zeiss Axioplan Microscope and recorded with a Hamamatsu Camera. **(A,E)** Well-populated B cells follicles with small numbers of small proliferating B cells, small follicular dendritic cell (FDC) networks, and intrafollicular CD8 T cells are appreciated in prostatic intraepithelial neoplasia (PIN) prostatectomy specimens. **(B,F)** Large PCNA^+^CD20^+^ B blasts, and CD8 T cells interspersed inside B cell follicles are detected in prostate samples from patients at intermediate stages of cancer. **(C,G)** A germinal center with a concentric FDC network, containing multiple large proliferating B blasts and surrounded by CD8 T cells was found inside some TLO of prostatectomy specimens from patients afflicted by advanced cancer. **(D,H)** Although proliferating B cells are notably reduced, B cell and CD8 T cells are still organized in TLO from evanescent prostate cancer patients. Yellow arrows point to proliferating B cells, while white arrow is depicting a proliferating CD8 T cell. Scale bars represent 100 μm.

It has been previously reported that the presence of TLO with dense B cell follicles and enriched with CD8 T cells correlates with favorable prognosis in cancer patients (18, 19). Thus, we first analyzed the cellular composition in tumor-associated TLO by staining prostatectomy specimens with antibodies specific for PCNA, CD8, and CD20. We also stained serial sections with antibodies against CD21 to visualize FDC and to get an idea of the effect of activated B cells on stromal cells. We found that prostate tissues from patients with PIN and prostate cancer have CD20^+^ B cells follicles with central FDC networks, which were surrounded by T cells areas populated by CD8 T cells (Figures 3A–H). Consistent with previous reports (18); we confirmed that TLO were very heterogeneous, even in the same prostate section. In PIN and intermediate prostate cancer, B cell follicles were loosely organized and contained few proliferating B cells (Figures 3A,B). In agreement with poor B cell activation, FDC networks were small in prostate tumors from patients with PIN and intermediate cancer (Figures 3E,F). Interestingly, some TLO in prostate tumors from patients with advanced disease contained many PCNA^+^CD20^+^ B cells (Figure 3C) and large CD21^+^ FDC networks (Figure 3G), indicating that despite the suppressive environment, activated B cells were still able to induce the differentiation of tumor-associated stromal cells into FDC. By contrast, prostate sections from patients with evanescent carcinoma had less PCNA^+^CD20^+^ B cells (Figure 3D) and contained well-organized FDC networks (Figure 3H). We observed similar staining pattern with Ki67 and PCNA in consecutive serial sections of inflamed tonsil and confirmed that in certain areas of the prostate tumor there was a permissive microenvironment, which supported immune cell proliferation (Figure S1 in Supplementary Material). Thus, it is likely that either local release of immunogenic prostate antigens (28) or immunologically permissive environments in certain areas of prostate tumors might still support local immune cell activation and thus contribute to stabilize TLO.

Previously, we showed that IL17 is critical at early stages of TLO formation in lungs from neonate mice instilled with LPS (10). Therefore, we decided to determine whether T cell-derived IL-17 participates in the formation of TLO in prostate cancer. Even though we did not find CD3^+^IL17^+^ T cells around TLO, we occasionally observed a few CD3^−^IL17^+^ cells outside of B cell follicles in prostatectomy specimens from patients with evanescent carcinoma. According to their location, they are probably IL17-producing type 3 innate lymphoid cells, which have been previously detected in close association to TLO in human lung cancer (29).

**Figure 4:**
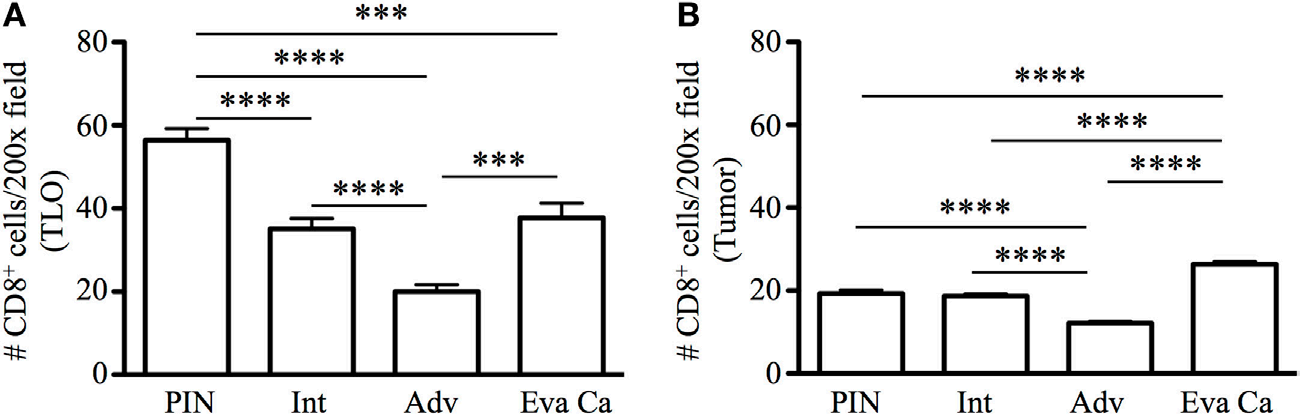
**Enumeration of CD8 T cells in tertiary lymphoid organs (TLO) and tumor areas of prostate cancer**. CD8 T cells were counted in all TLO contained in individual prostatectomy specimens or in five to eight randomly selected tumor areas lacking TLO (200× magnification). **(A)** Average number of CD8 T cells inside TLO and **(B)** average number of CD8 T cells in tumor regions are shown. *n* = 10–19 measurements/patient cohort. Bars represent mean ± SEM. Statistically significant differences (***p ≤ 0.0005, ****p < 0.0001) were calculated by using two-tailed Student’s *t*-test with GraphPad Prism.

Next, we enumerated CD8 T cells in TLO and tumor areas. Of note, CD8 T cells were approximately three times more numerous in TLO areas, compared to sites of malignant transformation (Figures 4A,B). Morphometric analysis revealed that CD8 T cells in TLO were significantly more abundant in PIN patients (56.45 ± 9.19), compared to numbers of CD8 T cells in TLO from intermediate (35.16 ± 8.53, *p* < 0.0001), advanced (20 ± 5.41, *p* < 0.0001), and evanescent prostate carcinoma (37.77 ± 10.61, *p* = 0.0005) (Figure 4A). Remarkably, CD8 T cells were significantly increased in TLO of evanescent carcinoma tumors, compared to CD8 T cells in TLO from advanced carcinoma patients (*p* = 0.0002). Furthermore, it was clear that CD8 T cells were considerably enriched in tumor areas of evanescent carcinoma patients, relative to CD8 T cell numbers in PIN (19.18 ± 2.4, *p* < 0.0001), intermediate (18.07 ± 1.07, *p* < 0.0001), and advanced prostate carcinoma (12.35 ± 1.27, *p* < 0.0001) (Figure 4B). Consistently, CD8 T cells were significantly reduced in TLO and tumor areas from patients at advanced stages of prostate cancer. Our results are indicating that spontaneous tumor regression in evanescent prostate cancer patients might be associated with enhanced ability of TLO to prime, recruit, or retain CD8 T cells (18, 19).

### Changes in Neovascularization and Cyclooxygenase 2 in TLO and Prostate Tumor Areas

**Figure 5:**
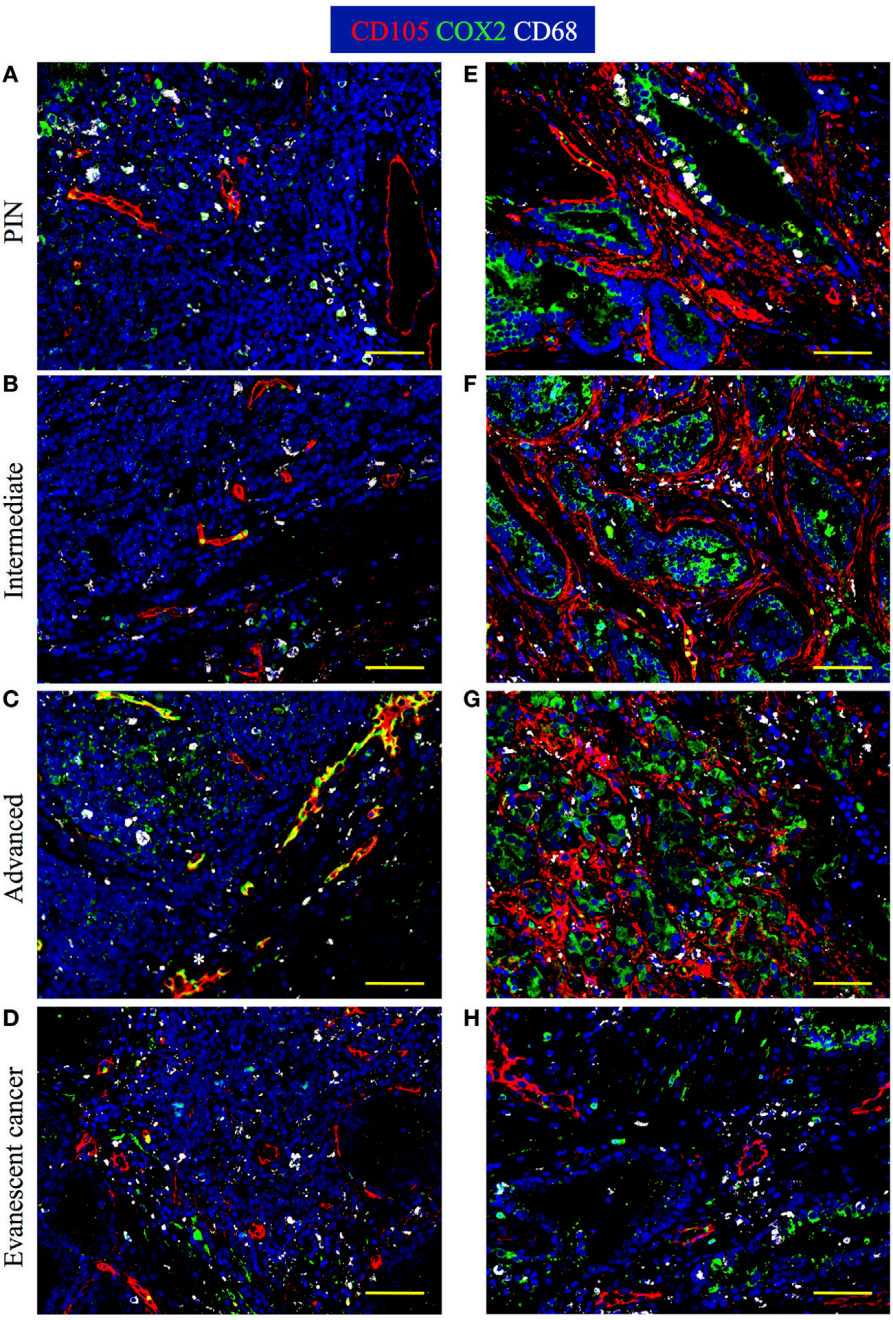
**Spatiotemporal changes in tumor vascularization and COX2 coverage at different stages of prostate cancer progression/regression**. The 5-μm thick paraffin serial sections were stained with antibodies against endoglin (CD105, red), cyclooxygenase 2 (COX2, green), and CD68 (white). **(A–D)** Representative 200× pictures are showing changes in vascularization and COX2 expression in tertiary lymphoid organs (TLO), or **(E–H)** tumor areas. **(A)** CD105^+^ vessels, CD68^+^COX2^+^ macrophages, and CD68^−^COX2^+^ cells are detected in TLO from prostatic intraepithelial neoplasia (PIN) patients. **(B)** Smaller CD105^+^ vessels and CD68^−^COX2^+^ cells are located on the border of TLO from intermediate prostate cancer patient. **(C)** In a prostatectomy specimen from advanced carcinoma patient, CD105^+^ vessels with cuboidal morphology are located outside TLO, while numerous CD68^−^COX2^+^ cells are found in the center of a TLO. **(D)** Oval-shaped and spindle-shaped COX2^+^ cells are located on the border of a TLO. **(E)** Vascularity and COX2^+^ epithelial cells are modestly increased in tumor areas from PIN patients. **(F,G)** Abundant blood vessels with abnormal morphology and aberrant organization were located in close proximity to strongly labeled COX2^+^ transformed epithelium in tumors of intermediate and advanced prostate cancer patients. **(D,H)** Preserved vascular morphology and organization, as well as reduced COX2^+^ density were observed in TLO and tumor areas from patients with spontaneous prostate cancer regression. CD105^+^ vessels with a high endothelial venule-like morphology were detected in TLO from patients at late stages of prostate cancer (white asterisk). Representative 200× magnification pictures of TLO and tumor areas were taken with a Zeiss Axioplan Microscope and recorded with a Hamamatsu Camera. Scale bars represent 100 μm.

It is known that tumors induce angiogenesis to grow and metastasize to distal organs (30–32). Based on the relevance of blood vessels in tumor growth, we evaluated the percentage of area covered by tumor vasculature in PIN and prostate cancer samples by immunofluorescence. Prostate sections were stained with antibodies specific for CD105 (endoglin), which is currently considered a good marker for proliferating endothelial cells and newly forming tumor vessels (33–36). Our immunofluorescence stain showed oval and cuboidal shaped endothelial cells in TLO vessels, indicating CD105 antibodies are labeling both blood vessels and high endothelial venules (HEV). In PIN samples, a few CD105^+^ HEV-like vessels were located in the center of TLO (Figure 5A), while in a few tumor areas, CD105^+^ vessels displayed an abnormal morphology and disorganized pattern (Figure 5E). In TLO areas, CD105^+^ HEV were small and less abundant (Figure 5B), contrasting with the increased vascularization seen in multiple tumor areas from intermediate carcinoma patients (Figure 5F). Unexpectedly, we still found CD105^+^ HEV in TLO from patients with advanced carcinoma, but they were small and have a flat appearance (Figure 5C). At a glance, CD105^+^ vessels in tumor areas looked similarly abundant in prostatectomy specimens from intermediate and advanced prostate cancer (Figures 5F,G). Finally, the area covered by CD105^+^ vessels was comparable in TLO and tumor areas from patients with evanescent carcinoma, and the blood vessel morphology and spatial arrangement were better preserved in both micro anatomical locations (Figures 5D,G).

Cyclooxygenase 2 (COX2) is an inducible enzyme that participates in the production of prostaglandin E_2_—a bioactive lipid with multiple immunomodulatory properties (37). In cancer, COX2 plays an important role on tumor-associated immune suppression, tumor-driven angiogenesis, and proliferation/renewal of cancer stem cells (38–40). Thus, we analyzed the morphology, localization, and distribution of COX2-producing cells in areas containing TLO (Figures 5A–D) and tumor areas (Figures 5E–H). According to their cell morphology and spatial location, we found that COX2 was expressed by a variety of tumor-infiltrating cells including cuboidal epithelial cells (Figures 5E–H), oval-shaped endothelial cells (Figures 5C,D), spindle-shaped fibroblasts, and immune cells. In TLO areas, COX2 antibodies labeled CD68^+^ macrophages (Figures 5A,C) and CD68^−^ immune cells inside B cell follicles (Figure 5C). It is likely that some of the CD68^−^COX2^+^ cells could be B cells, monocytes or monocyte-derived myeloid suppressor cells (41, 42). Sporadically, we also found COX2^+^ cells with hypersegmented nuclei morphology, which is typical for neutrophils or neutrophil myeloid-derived suppressor cells (43, 44).

**Figure 6:**
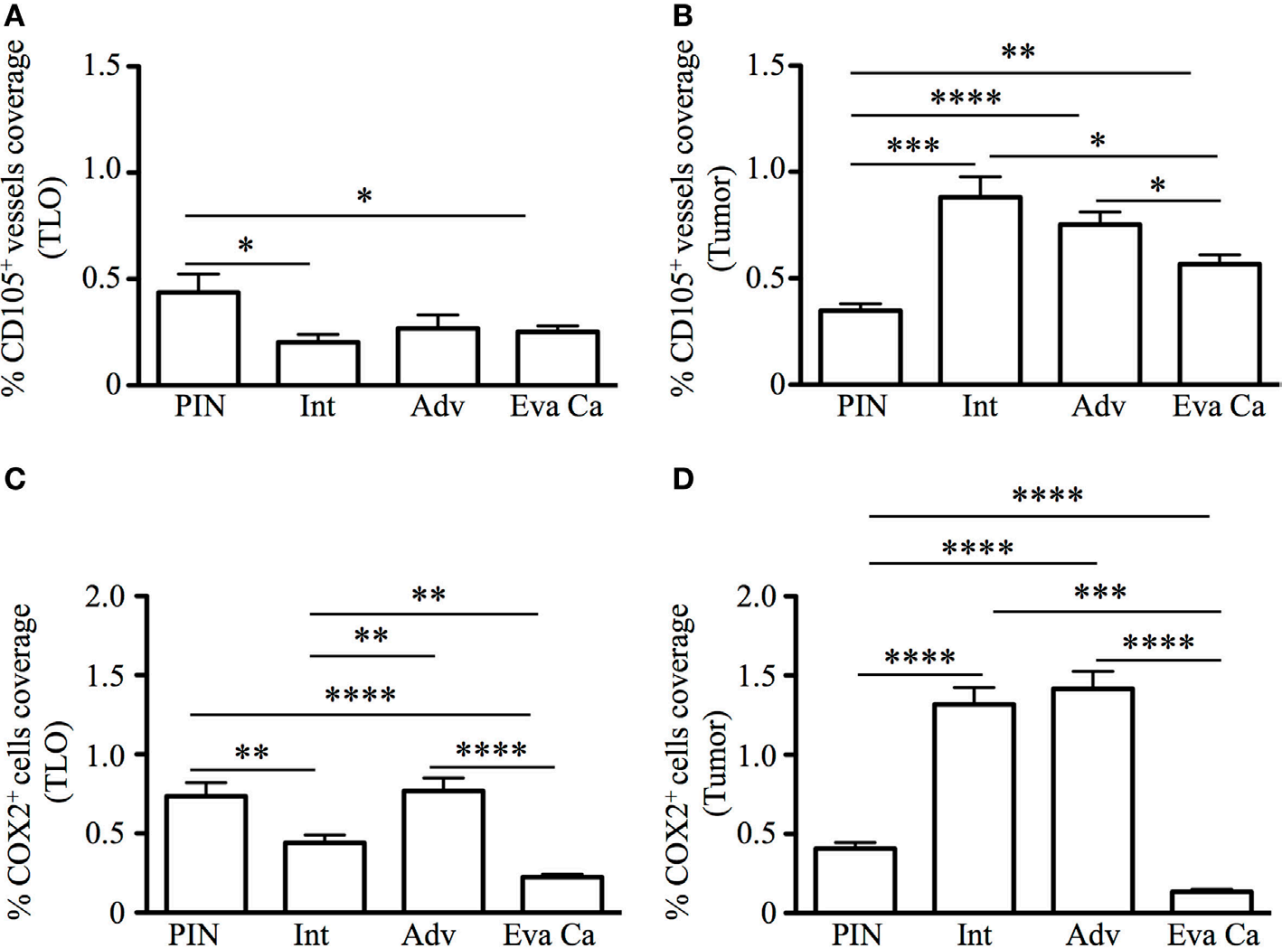
**Percentage of CD105^+^ blood vessel and COX2^+^ cell coverage during prostate cancer progression and regression**. To calculate the percentage of CD105^+^ vessel and COX2^+^ cell coverage in areas with tertiary lymphoid organs (TLO), lymphocytic structures were placed in the center, and 3 × 3 mosaic pictures were taken with the Zeiss Axioplan microscope (1.043 mm^2^). Panoramic mosaic pictures were taken for all the TLO contained in individual prostatectomy specimens. In areas lacking TLO and containing transformed epithelium, we measure the density for CD105^+^ vessels and COX2^+^ cells in three to five random fields/prostatectomy. **(A)** The percentage of CD105^+^ blood vessel coverage was smaller in prostate cancer and evanescent carcinoma, compared to prostatic intraepithelial neoplasia (PIN). **(B)** In tumor areas, percentage of CD105^+^ vessel coverage was the highest at intermediate and advanced stages of prostate cancer, compared to their percentage of coverage in PIN and evanescent prostate carcinoma samples. *n* = 7–35 panoramic mosaics/patient cohort to calculate CD105^+^ vessel coverage. **(C)** In TLO, percentage of COX2^+^ cell coverage was higher in advanced prostate carcinoma, compared to area covered by COX2^+^ cells in samples from intermediate and evanescent prostate carcinoma patients. **(D)** Areas occupied by COX2^+^ cells were significantly larger at intermediate and advanced stages of prostate cancer, while area covered by COX2^+^ cells was considerably smaller in evanescent prostate carcinoma, even when compared to percentage of area covered by COX2^+^ cells in PIN samples. *n* = 7–34 panoramic mosaic/group to calculate COX2^+^ cell coverage. Differences between groups were calculated by using two-tailed, paired, or unpaired Student’s *t*-test, using GraphPad Prism. Bar represent mean ± SEM. Statistically significant differences: *p ≤ 0.05, **p ≤ 0.005, ***p ≤ 0.0005, ****p < 0.0001.

Next, we calculated percentage of coverage by CD105^+^ blood vessels and COX2^+^ cells in TLO-associated microenvironments and tumor areas with equal dimensions, as explained in Section “Materials and Methods.” The percentage of area covered by CD105^+^ vessels was approximately two times larger in TLO from PIN patients (0.43 ± 0.33%), compared with area occupied by CD105^+^ vessels in TLO from intermediate (0.2 ± 0.16%, *p* = 0.0102) and evanescent carcinoma patients (0.25 ± 0.11%, *p* = 0.0401) (Figure 6A). In comparison to PIN (0.34 ± 0.12%), areas occupied by CD105^+^ vessels in regions of epithelial cell transformation were significantly larger in prostate sections from intermediate (0.88 ± 0.52%, *p* = 0.0006), advanced (0.75 ± 0.15%, *p* < 0.0001), and evanescent prostate carcinoma patients (0.56 ± 0.16%, *p* = 0.0016) (Figure 6B).

In addition, we found that percentage of area covered by COX2^+^ cells in TLO was significantly smaller in prostatectomy specimens from evanescent carcinoma patients (0.22 ± 0.07%), compared to areas occupied by COX2^+^ in prostate sections from PIN (0.73 ± 0.27%, *p* < 0.0001), intermediate (0.44 ± 0.24%, *p* = 0.0017) and advanced carcinoma patients (0.76 ± 0.18%, *p* < 0.0001) (Figure 6C). Percentage of COX2^+^ cell coverage in tumors from evanescent prostate carcinoma was even significantly smaller than percent of area covered by COX2^+^ cells in PIN and prostate cancer samples (0.13 ± 0.06, *p* < 0.0001 vs all patient cohorts). In tumor areas, percentage of coverage by COX2^+^ cells was significantly higher in intermediate (1.31 ± 0.51%, *p* < 0.0001) and advanced prostate carcinoma (1.41 ± 0.31%, *p* < 0.0001), compared to PIN (0.40 ± 0.13%). (Figure 6D). Overall, our results show that COX2 is an important player in tumor-driven suppression, which is likely impairing TLO-derived protective immunity during cancer progression.

### PD-L1^+^ Inflammatory Cells Accumulate Preferentially at Tumor Areas in during Prostate Cancer Progression

**Figure 7:**
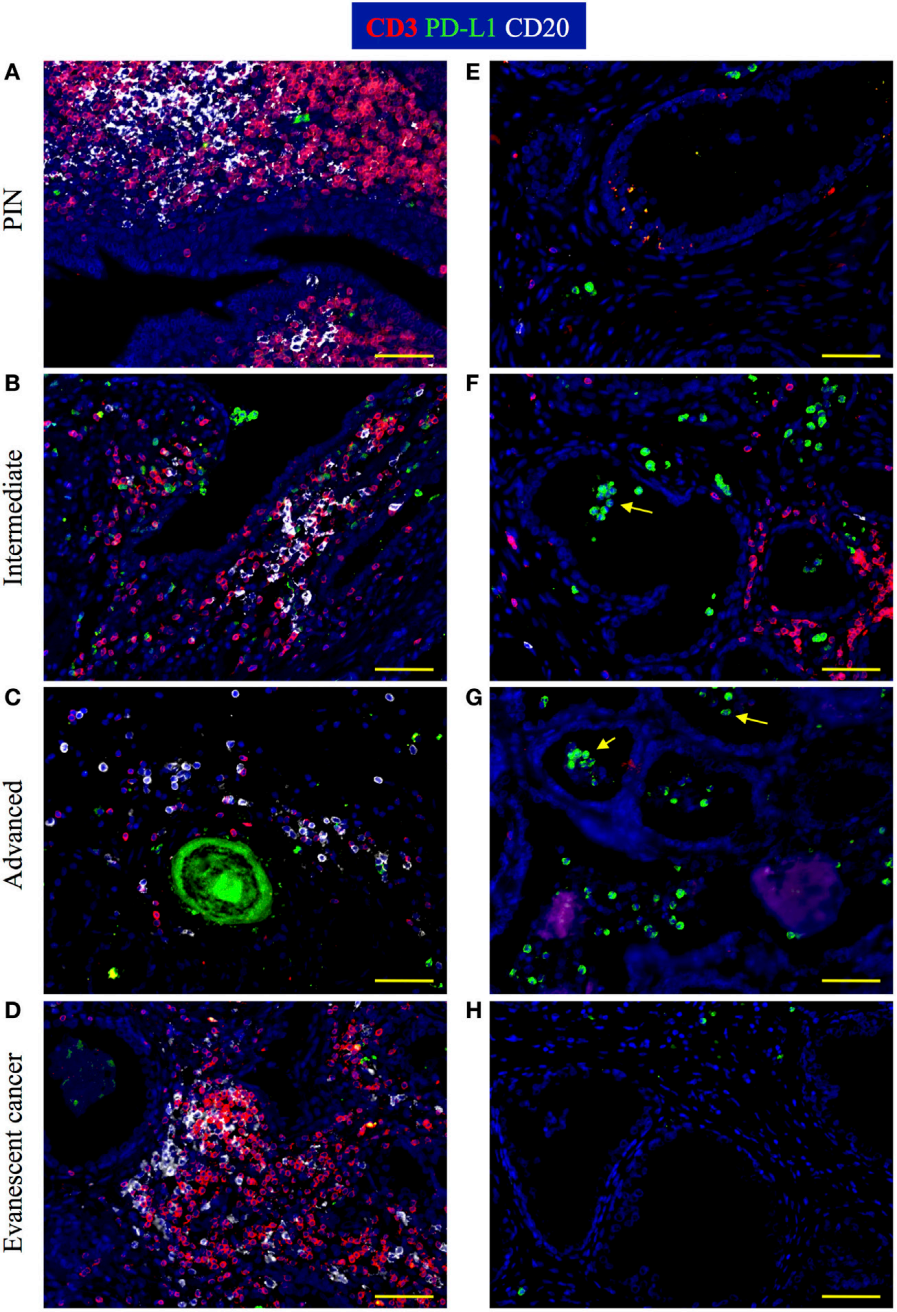
**Visualization of PD-L1 expression during prostate cancer progression and cancer regression**. Prostate sections were stained with antibodies against CD3, PD-L1, and CD20. **(A)** In prostatectomies from patients with prostatic intraepithelial neoplasia (PIN), PD-L1 was expressed by a few cells intermixed with T cells. **(B,C)** At intermediate and advanced stages of prostate cancer, PD-L1^+^ cells were more numerous and were closely interacting with T cell and B cells. **(D)** PD-L1^+^ cells were scarce in tertiary lymphoid organs (TLO) from patients with evanescent prostate carcinoma. **(E)** Few PD-L1^+^ tumor-infiltrating cells are located in close proximity to epithelium in a prostatectomy from a patient with PIN. **(F)**. Although there is still accumulation of T cells close to glandular epithelium, more PD-L1^+^ cells accumulated in tumor areas of intermediate prostate cancer. **(G)** Considerable accumulation of PD-L1^+^ cells and lack of T cell infiltrates are seen in tumor areas of prostatectomies from advanced cancer patients. **(H)** A few cells positive for PD-L1 are detected in close proximity to glandular epithelium in samples from evanescent prostate carcinoma patients. Representative 200× magnification pictures from TLO and tumor areas were taken with a Zeiss Axioplan Microscope and recorded with a Hamamatsu Camera. Yellow scale bar represents 100 μm. Yellow arrows are pointing to PD-L1^+^ cells with polymorphonuclear-like morphology.

It was recently discovered that PGE_2_ induces PD-L1 expression on myeloid suppressor cells contributing to local tumor immunosuppression. Thus, we decided to analyze changes in PD-L1 expression during prostate cancer progression or regression. We detected PD-L1^+^ cells in TLO areas from patients with PIN and from prostatectomies collected at different stages of prostate cancer progression (Figures 7A–C). By contrast, TLO from patients with evanescent prostate carcinoma contained less PD-L1^+^ cells (Figure 7D). In tumor areas, we detected few PD-L1^+^ cells in prostatectomies from PIN (Figure 7E) and evanescent prostate carcinoma samples (Figure 7H). It was evident that PD-L1^+^ were more numerous in tumor areas of patients affected by intermediate and advanced prostate cancer (Figures 7F,G). Thus, it seems that prostate tumor microenvironment is either attracting suppressive cells that express PD-L1 or inducing the expression of PD-L1 in tumor-infiltrating cells.

### Dynamic Changes in CD3^+^Tbet^+^ Th1 Cells and CD3^+^Foxp3^+^ Treg Inside TLO Correlate with Cancer Progression and Spontaneous Prostate Cancer Regression

**Figure 8:**
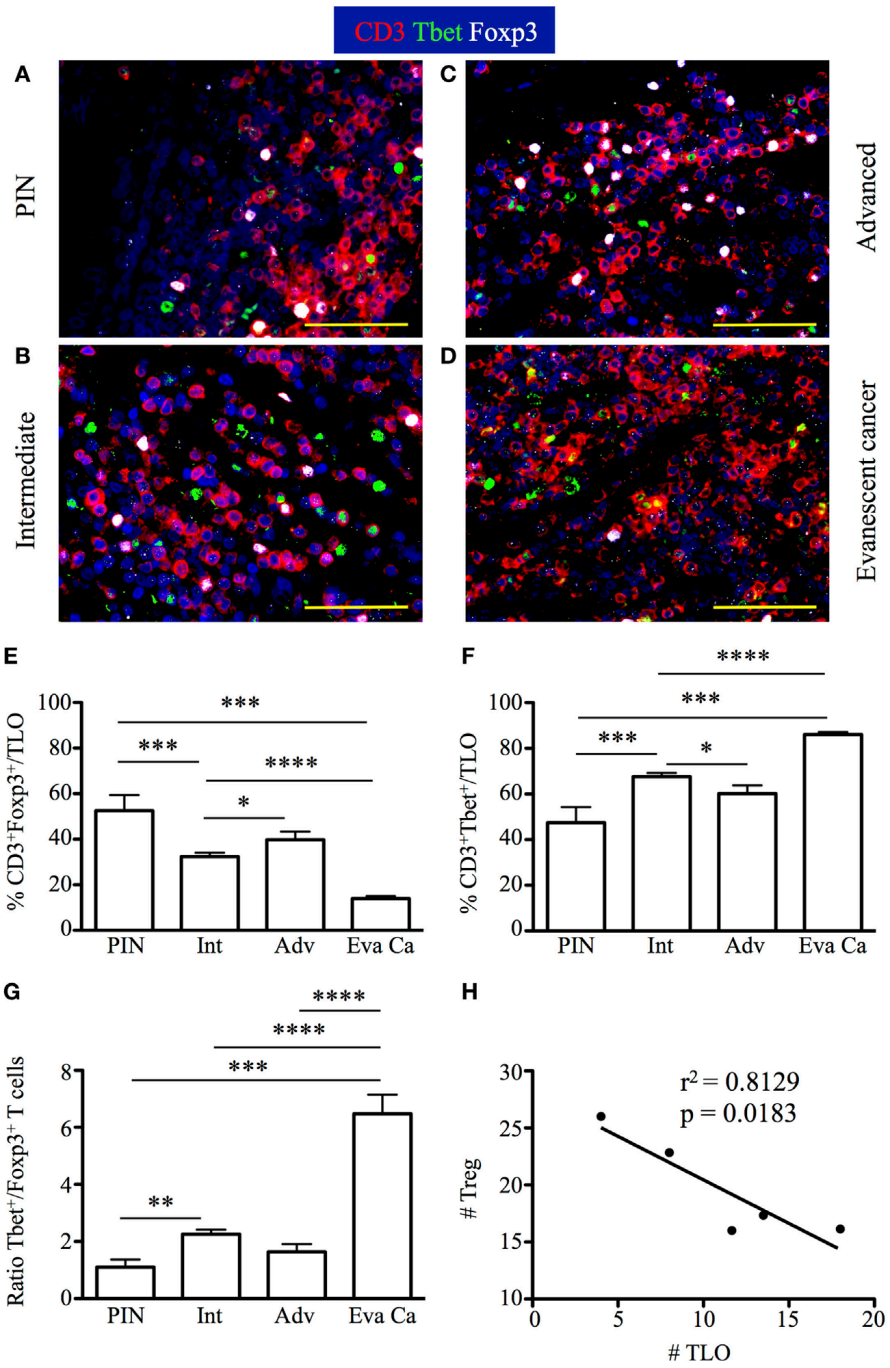
**Increased accumulation of regulatory T cells (Treg) in tertiary lymphoid organs (TLO) correlate with cancer progression, while drastic enrichment in Tbet^+^ T cells inside TLO coincides with cancer regression**. The 5-μm thick paraffin sections were stained with antibodies against CD3 epsilon, Tbet, and Foxp3 to identify CD3^+^ T cells, CD3^+^Tbet^+^ Th1 cells, and CD3^+^Foxp3^+^ Treg inside TLO. Representative 400× pictures were taken with a Zeiss Axioplan microscope and recorded with a Hamamatsu camera. **(A–C)** Nuclear Foxp3 (white) and Tbet stain (green) of CD3 T cells (red) reveal a visually apparent overrepresentation of CD3^+^Foxp3^+^ T cells inside TLO of advanced prostate carcinoma patients. **(D)** Few Treg intermixed with considerable amounts of CD3^+^Tbet^+^ T cells are detected in dense T cell areas of TLO from evanescent carcinoma patients. Scale bar represents 100 μm. **(E)** Percentage of Foxp3^+^ Treg and **(F)** CD3^+^Tbet^+^ in TLO. **(G)** Ratio between Tbet Th1 cells and Treg was considerably and significantly increased after spontaneous prostate cancer regression. **(H)** Significant correlation between number of Treg and number of TLO was determined by Pearson’s coefficient. Treg inside TLO inversely correlated with a decrease in the number of TLO (*r*^2^ = 0.8129, *p* = 0.0183). *n* = 6–22 200× fields were used to enumerate Tbet^+^ T cells and Foxp3^+^ Treg per patient cohort. Differences between groups were calculated by using two-tailed, paired or unpaired Student’s *t*-test, using GraphPad Prism. Bar represent mean ± SEM. Statistically significant differences: *p ≤ 0.05, **p ≤ 0.005, ***p ≤ 0.0005, ****p < 0.0001.

Tertiary lymphoid organs induce protective immunity at peripheral locations (45–47). However, we were perplexed about the similar size of tumor-associated TLO in advanced and evanescent prostate carcinoma samples. Because PGE_2_ modulates Th1 immunity and support differentiation and functions of Treg—cells key in modulating inflammation, adaptive immunity, and TLO organization (48–52), we focused on detecting Tbet^+^ T cells and Foxp3^+^ Treg by immunofluorescence in prostatectomy specimens. We observed that CD3^+^Foxp3^+^ and CD3^+^Tbet^+^ T cells accumulated in T cell areas of TLO, and even we could occasionally find Th1 and Treg establishing close interactions (Figures 8A–C). Massive accumulation of CD3^+^Tbet^+^ T cells was obvious in TLO from evanescent prostate carcinoma patients (Figure 8D).

Consistent with our predictions, the percentage of CD3^+^Foxp3^+^ T cells was higher in TLO from advanced prostate carcinoma (22.83 ± 7.83%) and PIN samples (24.4 ± 28.56%), compared to their frequencies in TLO from intermediate (15.28 ± 6.52%, PIN vs intermediate: *p* = 0.0287) and evanescent carcinoma patients (15.42 ± 5.02%) (Figure 8E). Conversely, higher percentage of CD3^+^Tbet^+^ cells was detected in TLO from evanescent carcinoma sections (98.3 ± 2.86%), compared to their percentage in TLO from PIN (23.57 ± 12.48%, *p* = 0.0001), intermediate (37.41 ± 8.35%, *p* < 0.0001) and advanced prostate carcinoma patients (34.33 ± 8.35%, *p* = 0.0080) (Figure 8F). CD3^+^Tbet^+^ T cells were enriched in TLO from evanescent prostate carcinoma patients, as shown by the significant increase in the ratio between Tbet^+^ and Foxp3^+^ T cells (6.47 ± 1.76), relative to PIN (1.1 ± 0.7, *p* = 0.0006), intermediate (2.25 ± 0.76, *p* < 0.0001), and advanced prostate carcinoma patients (1.63 ± 0.67, *p* < 0.0001) (Figure 8G). We also enumerated Treg and Tbet T cells in prostate parenchyma, around blood vessels, and in intraepithelial areas, but we did not find any trend between cancer stages and changes in the accumulation of Tbet^+^ or Foxp3^+^ T cells. We only detected a significant increase in the number of Treg in all tumor microenvironments of patients at advanced stages of prostate cancer (data not shown). Finally, there was a significant inverse correlation between the number of TLO and the number of Treg (*r*^2^ = 0.8129, *p* = 0.0183) (Figure 8H). Thus, our results are suggesting that numerical changes in Treg and Th1 cells inside TLO are more directly associated with prostate cancer progression or spontaneous regression and are likely showing that besides the role of Treg in modulating local inflammation and adaptive immunity, they are possibly affecting TLO organization during prostate cancer progression (51, 52).

### Impaired Accumulation of Granzyme B^+^ Cells Correlates with Reduced Numbers of Mature DC-LAMP^+^ Antigen-Presenting Cells (APC) and Small HEV in Advanced Prostate Carcinoma

**Figure 9:**
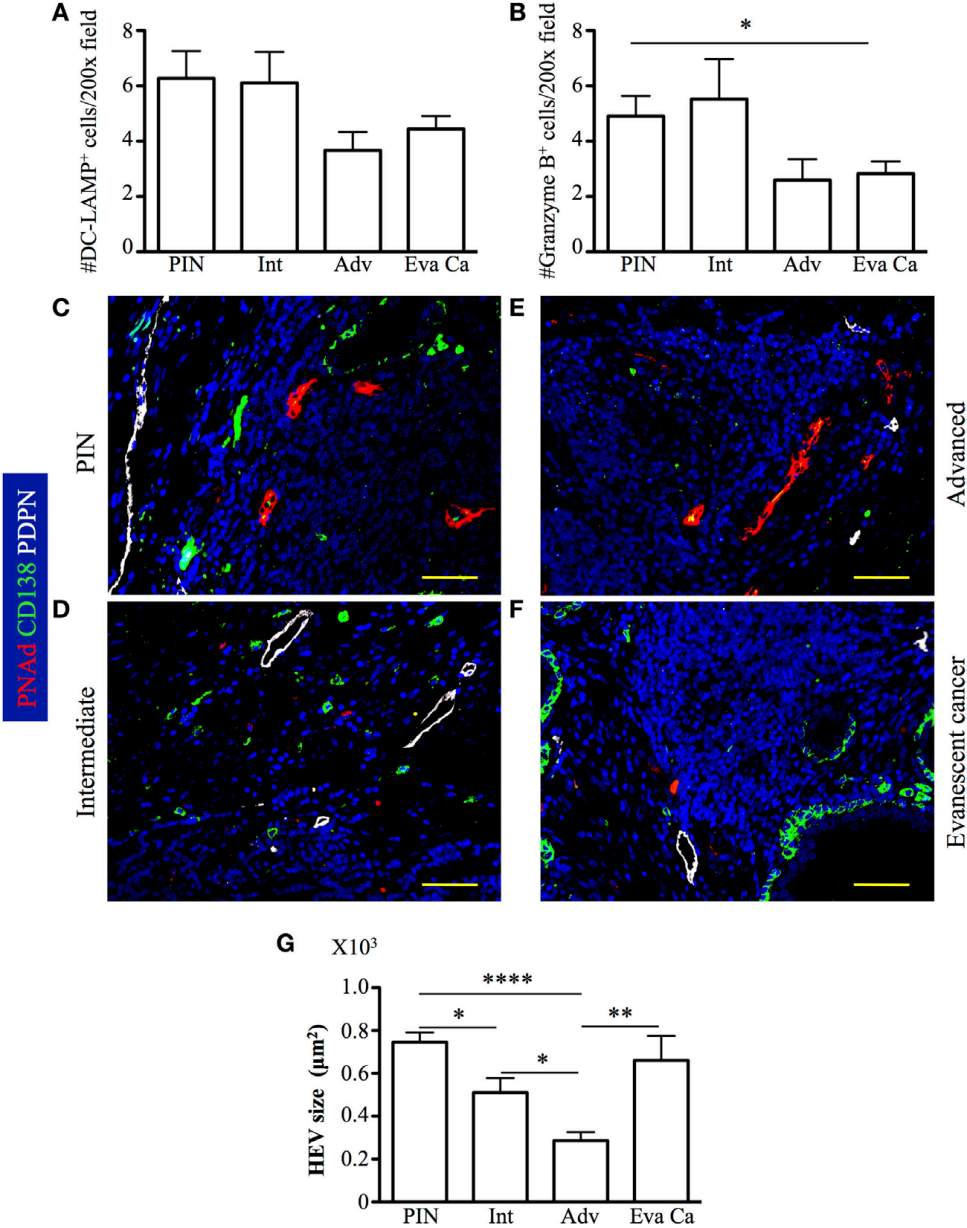
**Accumulation of DC-LAMP^+^ antigen-presenting cells (APC) and granzyme B^+^ cells is associated to reduction in the size of high endothelial venules (HEV) at late stages of prostate cancer**. DC-LAMP^+^ cells and granzyme B^+^ cells were enumerated in all the tertiary lymphoid organs (TLO) of individual prostatectomy specimens collected at different stages of prostate cancer progression/regression. **(A,B)** DC-LAMP^+^ APC and granzyme B^+^ cells were significantly reduced in advanced and evanescent prostate cancer. **(C–F)** Representative 200× pictures showing location of lymphatics stained with antibodies specific for podoplanin (PDPN, white vessels), HEV labeled with antibodies specific for peripheral node addressin (PNAd, red vessels), and CD138^+^ plasma cells and epithelium (green). **(G)** Progressive reduction in the size of HEV coincides with prostate cancer progression, while recover in HEV size is seen in TLO of patients with evanescent prostate cancer. DC-LAMP^+^ APC and granzyme B^+^ cells were enumerated in 5–19 200× fields. Scale bars represent 100 μm. Differences between groups were calculated with two-tailed, paired, or unpaired Student’s *t*-test, using GraphPad Prism. Bar represent mean ± SEM. Statistically significant differences: *p ≤ 0.05, **p ≤ 0.005, ****p < 0.0001.

To identify additional immune cells that are key in tumor immunity, we counted granzyme B^+^ CD8 T cells and mature dendritic cell lysosomal-associated membrane protein (DC-LAMP, CD208) APC inside TLO. We found increased numbers of mature DC-LAMP^+^ cells in PIN (6.27 ± 3.25), compared to their numbers in advanced (3.66 ± 1.63, *p* = 0.0445) and evanescent prostate carcinoma (4.44 ± 2.0, *p* = 0.0356) (Figure 9A). Numbers of granzyme B^+^ cells showed a similar trend, with significantly higher numbers in PIN (4.9 ± 2.42), compared to advanced (2.6 ± 1.67) and evanescent prostate carcinoma (2.83 ± 1.85, *p* = 0.0149) (Figure 9B). Unexpectedly, both populations were significantly reduced in prostatectomy specimens from patients with advanced and evanescent prostate carcinoma (Figures 9A,B). Thus, it is likely that poor accumulation of mature APC in TLO during advanced prostate carcinoma may be linked to impaired Th1 priming and compromised generation or recruitment of granzyme B^+^ cells, while their apparent reduction in evanescent carcinoma may reflect the contraction of the tumor immune response.

Although, the impact of Treg on HEV is controversial, some studies have proposed that Treg affect HEV differentiation (53, 54). Thus, given that we found a significant number of Treg in TLO from advanced prostate cancer patients, we hypothesized that HEV vasculature can be compromised in this particular cohort of cancer patients. We identified HEV and podoplanin^+^ lymphatic vessels in close proximity to B cell follicles (Figures 9C–F). The presence of lymphatics and HEV in PIN and at all stages of prostate cancer progression suggests that mature APC: naïve and central memory CD62L^+^ T cells can be recruited to tumor-associated TLO. It was also apparent that T cells were providing help to B cells because we were able to detect CD138^+^ plasma cells, which may be actively produced in tumor-associated TLO. Supporting the idea that Treg affect the integrity of HEV, we found that HEV were significantly smaller in prostatectomy specimens from advanced carcinoma patients (285.86 ± 148.40), compared to their size in intermediate prostate carcinoma samples (510.60 ± 314.76, *p* = 0.0178). Interestingly, HEV were significantly larger in TLO from patients with evanescent prostate carcinoma (660.74 ± 393.82, *p* = 0.0030), relative to HEV size in TLO from advanced carcinoma patients (Figure 9G). However, despite that we are proposing Treg are affecting HEV integrity, we cannot rule out that tumor microenvironment can affect the production of vascular endothelial growth factors by other immune cells (DC or B cells) and thus affect HEV growth (55, 56).

### Visual Estimation of Antigen Load in Prostate Tumors by Detection of PSCA

**Figure 10:**
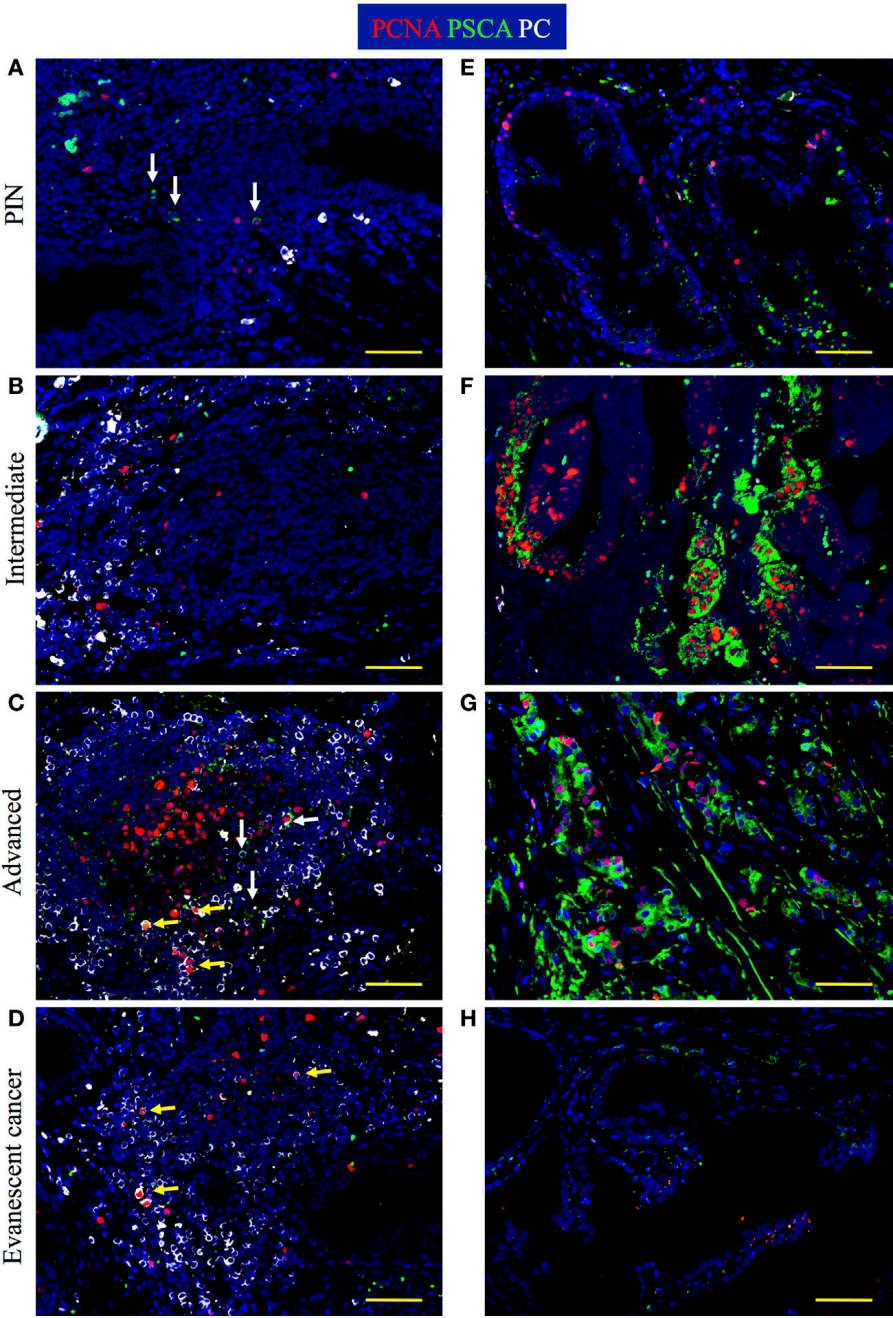
**Increased tumor antigen load and active production of plasma cells are found at late stages of prostate cancer**. The 5-μm thick paraffin sections were stained with antibodies against proliferating cell nuclear antigen (PCNA) (red), prostate stem cell antigen (PSCA, green), and plasma cell antigen (PC, white) to visualize PSCA tumor antigen and plasma cell generation at different stages of cancer progression/regression. Representative 200× pictures were taken with a Zeiss Axioplan microscope and recorded with a Hamamatsu camera. **(A)** Cells with eccentric nuclei (white) and cells positive for PSCA are detected in tertiary lymphoid organs (TLO) of prostatic intraepithelial neoplasia (PIN) samples. **(B)** Plasma cells are detected on the margins of TLO from patients at intermediate stages of prostate cancer. **(C)** Active germinal center that contains proliferating plasma blasts, non-proliferating plasma cells, and PSCA^+^ cells was found in a sample from a patient at advanced stages of prostate cancer. **(D)** Proliferating plasmablasts are detected in a small TLO from a patient with evanescent prostate carcinoma. **(E)** Low proliferation and scarce PSCA signal in the epithelium of a PIN patient. **(F)** Highly proliferative but still organized epithelium in a tumor area of a patient at intermediate stage of prostate cancer. **(G)** Disorganized and proliferating nests of epithelial cells in a tumor area of patient with advanced prostate cancer. **(H)** PSCA signal was drastically reduced and epithelium had minimal evidence of proliferation in samples from patients with evanescent prostate cancer. Yellow arrows are pointing to proliferating plasmablasts that are indicative of local antigenic stimulation, while white arrows are depicting PSCA^+^ cells. Scale bar represents 100 μm.

To test whether reduction of granzyme B^+^ cells correlate with changes in tumor antigen load in TLO from advanced and evanescent carcinoma patients, we stained prostate sections with antibodies against PCNA, PSCA, and plasma cell antigen. In lesions from PIN patients, PSCA^+^ cells inside TLO were rare and a few of them had a monocytic morphology (Figure 10A), while PSCA^+^ epithelial cells were scarce and were detected in a few tumor areas (Figure 10E). PSCA^+^ cells were not detectable in TLO (Figure 10B), and proliferating epithelial cells labeled with PSCA antibodies were detected in some tumor areas from intermediate prostate cancer patients (Figure 10F). In patients with advanced prostate cancer, few PSCA^+^ cells were inside TLO (Figure 10C), contrasting with the considerable numbers of proliferating PSCA^+^ transformed epithelial cells in tumor areas (Figure 10G). As expected, PSCA^+^ cells were almost absent in TLO and tumor areas of samples with evanescent carcinoma (Figures 10D,H). Morphometric analysis confirmed our observations and revealed a significantly bigger area covered by PSCA in panoramic pictures of prostatectomies from intermediate (830,261.83 ± 267,703.70, *p* = 0.0020) and advanced prostate cancer patients (693,380.42 ± 127,344.12, *p* = 0.0002), compared to PIN (158,207.25 ± 155,598.37). In sharp contrast, PSCA area was significantly reduced in evanescent carcinoma (80,452 ± 36,933.11), compared to the area covered by PSCA in intermediate (*p* = 0.0023) and advanced prostate cancer prostatectomies (*p* < 0.0001) (Figure S2 in Supplementary Material). To confirm that B cells were activated in TLO, prostatectomy specimens were stained with antibodies specific for plasma cells. Immunofluorescence images revealed the presence of large cells with eccentric nuclei in all prostatectomy specimens (Figures 10A,D). Even we found a few proliferating plasma cells, which are possibly plasmablasts that were recently produced in germinal centers. Altogether, our results suggest that antigenic stimulation is taking place in selected tumor locations during cancer progression, and that reduction in the antigenic load might be linked to destabilization of TLO in patients with evanescent prostate carcinoma.

### CXCL10 Production by Immune Cells in TLO Is Critical for Mediating Control of Tumor Cells in TLO Areas

**Figure 11:**
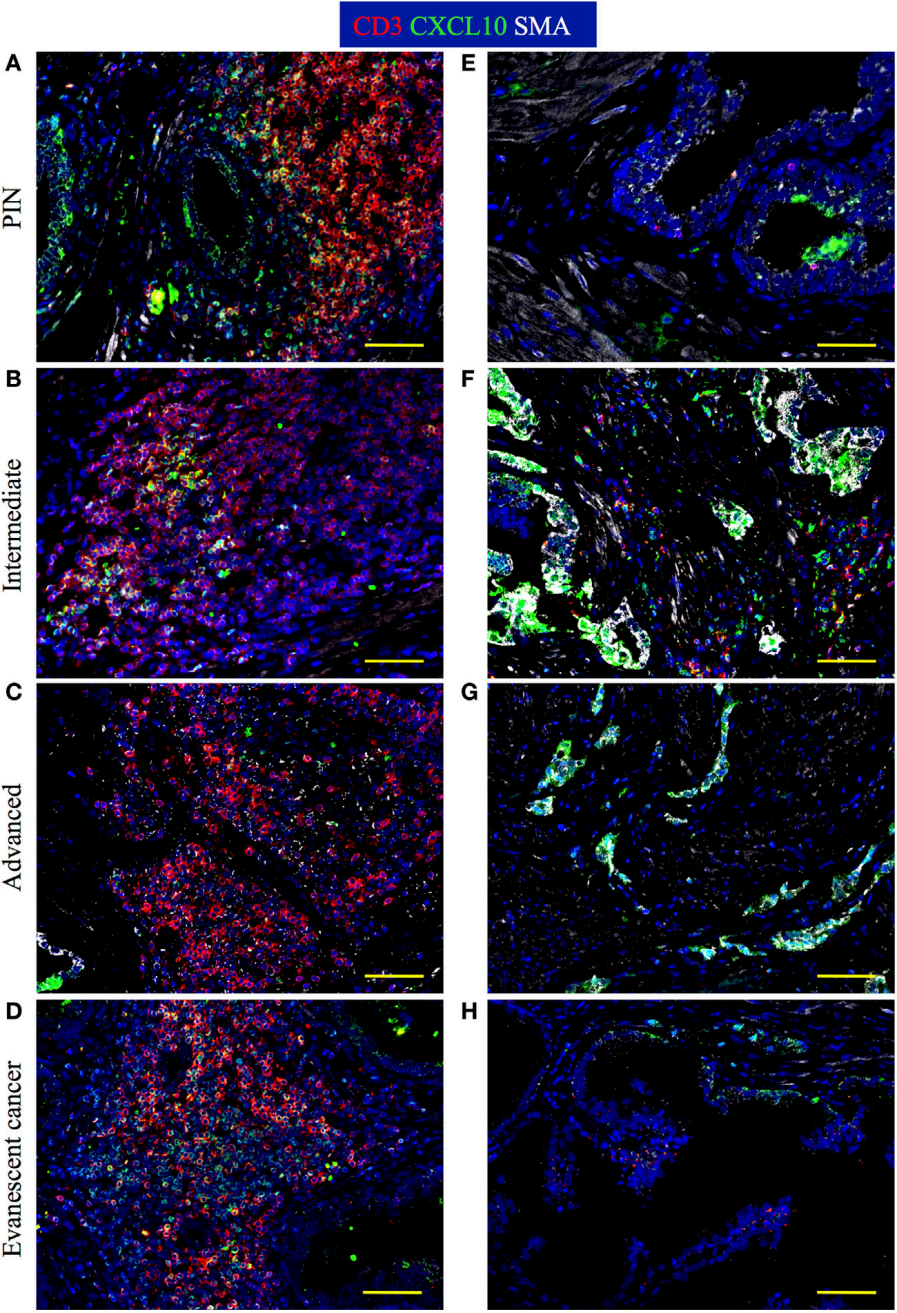
**Immune cells are positive for CXCL10 stain inside tertiary lymphoid organs (TLO), while CXCL10^+^ epithelial cells are found at tumor areas**. The 5-μm thick paraffin sections were stained with antibodies against CD3 epsilon (red), CXCL10 (green), and smooth muscle actin (white). Representative 200× pictures were taken with a Zeiss Axioplan microscope and recorded with a Hamamatsu camera. **(A)** CXCL10 mainly labels CD3^+^ T cells inside TLO of prostatic intraepithelial neoplasia (PIN) prostatectomy specimens. **(B)** CXCL10^+^CD3^+^ T cells are reduced in TLO of patients with intermediate prostate cancer. **(C)** Immunosuppression in advanced prostate cancer correlates with a drastic reduction in CXCL10^+^CD3^+^ T cells inside TLO. **(D)** CD3^+^ and CD3^−^CXCL10^+^ cells are notably increased in small TLO of patients with evanescent prostate carcinoma. **(E)** CXCL10^+^ epithelial cells are sporadically found in prostatectomy specimens from PIN. **(F,G)** Intense CXCL10 signal is mainly detected in transformed epithelial cells at intermediate and late stages of prostate cancer. **(H)** CXCL10^+^ epithelial cells are rarely detected in tumor areas of patients with evanescent prostate carcinoma. Scale bar represents 100 μm.

Attraction of lymphocytes to TLO is orchestrated by homeostatic chemokines (CXCL13, CCL19, CCL21) produced by stromal cell populations in the T and B cell zones (4, 5, 57–59). However, based on the increased numbers of Tbet^+^Th1 cells in TLO from patients with evanescent prostate carcinoma, we expected that IFNγ, produced by Th1-cells, stimulates local production of CXCL10—a chemokine that attracts effector lymphocytes to inflammatory sites (60). Thus, we decided to stain sections with antibodies against CD3, CXCL10, and smooth muscle actin to define the location of CXCL10-producing cells in TLO and tumor areas. In PIN samples, we found CD3^+^ T cells and CD3^−^ immune cells positive for CXCL10 in TLO (Figure 11A). Consistent with previous reports (61, 62), CXCL10^+^ epithelial cells were detected in a few tumor locations in PIN specimens (Figure 11E). At intermediate stages of prostate cancer, a considerable number of CXCL10^+^CD3^+^ T cells were located inside TLO (Figure 11B), and multiple epithelial cells were positive for CXCL10 in tumor areas (Figure 11F). In advanced prostate carcinoma and consistent with the reduction in Tbet^+^ Th1 cells, we noticed a reduction in CXCL10^+^CD3^+^ T cells in TLO (Figure 11C) but unexplainably observed intense CXCL10 staining in the transformed glandular epithelium (Figure 11G). Finally, we found many CD3^+^ and CD3^−^CXCL10^+^ cells in the few and small TLO from patients with evanescent prostate carcinoma (Figure 11D), contrasting with the scarce and weak expression for CXCL10 in the glandular epithelium (Figure 11H). Area covered by CXCL10^+^ epithelial cells was significantly larger in intermediate (453,886.16 ± 177,813.12, *p* = 0.0031) and advanced prostate cancer patients (713,643.42 ± 242,242.98, *p* = 0.0003), compared to patients with PIN (102,605.8 ± 89,134.77) or evanescent carcinoma (64,537.8 ± 52,063.40, Eva Ca vs Int: *p* = 0.0011, Eva Ca vs Adv: *p* = 0.0002) (Figure S2 in Supplementary Material). Our results suggest that the prostate possesses a unique environment that supports the local production of CXCL10, which is critical for the attraction of CXCR3^+^ effector cells to tumor-associated TLO.

## Discussion

Here, we show that immune cells infiltrating prostate tumors at different stages of cancer progression (PIN, intermediate, advanced, and evanescent carcinoma) were organized in complex lymphocytic structures that resemble mature TLO (FDC networks, HEV, lymphatics, B cell follicles, T cell areas, mature dendritic cells). Given that LTα_1_β_2_ expression on B cell and dendritic cells is critical for stromal cells differentiation into FDC and blood vessels into HEV (63–66), we are assuming that activated B cells and dendritic cells are responsible for the morphological changes in stromal cells inside tumor-associated TLO (FDC, HEV) and for stimulating the local production of homeostatic chemokines (CCL19, CCL21, CXCL13) that are key in the attraction and local activation of immune cells in TLO embedded in prostate tumors (67, 68).

Several studies have shown that TLO support protective immunity against a variety of pathogens (46, 47, 58) or cause tissue damage in inflammatory and autoimmune diseases (15, 69–72). Interestingly, presence of TLO in solid tumors correlated with a favorable clinical outcome (18, 19). That TLO are detected in aggressive tumors suggests that malignant cells are probably able to negatively modulate TLO-driven tumor immunity (25, 73). Consistent with this idea, our study shows that TLO are present at all stages of cancer progression (PIN, intermediate, advance, and evanescent carcinoma). This motivated us to perform a detailed analysis of tumor environment surrounding TLO with the goal of identifying cell or molecular targets that can lead to designing personalized therapies focused to enhance TLO functions in prostate cancer patients.

We observed a clear reduction in the number and size of LF in intermediate and advanced prostate carcinoma patients, which is potentially indicating that the tumor suppressive microenvironment is exerting a negative pressure on TLO formation, organization, and function. Surprisingly, even in advanced prostate cancer, we found a few TLO with clear evidence of local immune cell activation (FDC networks, HEV, B cell, and CD8 T cell proliferation). But the most exciting finding in our study was the reduction in COX2 cell coverage (TLO and non-TLO areas), accompanied by the low number of Treg and the considerable increase in Tbet^+^ Th1 cells in TLO from unique patients that experienced spontaneous prostate cancer regression. This drastic change in the cellular composition of tumor-associated TLO in patients with evanescent prostate cancer is totally opposite to many of the parameters associated with impaired survival in solid cancers (74, 75) and consistent with the positive prognostic value of tumor infiltration by Th1 cells (76, 77).

### Is the Vasculature an Appropriate Therapeutic Target in Prostate Cancer?

It is known that newly formed blood vessels provide nutrients, oxygen, and soluble factors for the growth of malignant cells. Indeed, multiple studies have shown that tumor progression is associated with a global increase in vascularity (78). Unexpectedly, we found a reduction in the density of CD105^+^ blood vessels in microenvironments around TLO during prostate cancer progression. By sharp contrast, chaotic and exuberant neovascularization was increased in areas dominated by transformed epithelial cells. Although several groups have proposed that CD105 should be targeted to eliminate tumor blood vessels, there is a potential risk for affecting CD105^+^ HEV and thus impairing recruitment of CD62L^+^ naïve and central memory cells into TLO. Poor recruitment of CD62L^+^ naïve T cells into the prostate tumor could drastically affect the local generation of tumor specific T cells that recognize neoantigens, which are produced in response to local immune pressure and play an important role in immune evasion. In addition, disruption of HEV functions will likely prevent recruitment of central memory T cells, which have been previously detected in the circulation of cancer patients after administration of neoadjuvant therapies and that are equipped with potent cytokines that kill tumor cells (79). However, it is difficult to know whether HEV and lymphatics are providing a functional navigation system for APC and T cells inside prostate tumors or whether those specialized vessels are establishing a productive communication with draining lymph nodes. Thus, it is encouraging to think that instead of targeting the vasculature, we should take advantage of HEV and lymphatics already present in prostate tumor-associated TLO to deliver antigen-loaded mature dendritic cells and/or CD62L^+^ T cells, which will probably accelerate the elimination of malignant prostate cells.

### Delicate Balance among Antigen Load, Inflammation, and Immunosuppression Dictates the Fate of TLO Organization and Function

Several groups have proposed that chronic inflammation eventually leads to cancer (80–82). Earlier, Nancy Ruddle’s group proposed that chronic inflammation is the driving force behind TLO formation (7). Thus, it seems that TLO formation and organization cannot occur independently of cancer development. However, it is anticipated that the suppressive microenvironment in prostate tumors will impair TLO organization and functions. Consistent with the link between chronic inflammation and TLO formation, we observed bigger TLO in patients with PIN, compared to TLO in patients at intermediate and advanced stages of prostate cancer. Also, in agreement with previous reports (18), there was a considerable heterogeneity in the size and complexity of tumor-associated TLO in prostatectomy specimens. Heterogeneity in TLO may be simply associated to different phases of TLO organization. Alternatively, TLO size and cellular complexity may be linked to the local tumor antigen load. In our study, we found indirect evidence of lymphocyte activation by local antigens (proliferation of B cell, CD8 T cells, plasma cells) in certain areas of prostate tumors containing TLO. This possibly means that more immunogenic tumor antigens are released in those areas, stimulating lymphocytes and thus enhancing TLO organization. Alternatively, TLO organization can be affected by accumulation of suppressive cells (Treg, myeloid-derived suppressor cells, alternatively activated macrophages) in the surrounding microenvironment. In our study, this dichotomy was clearly exemplified in TLO from patients with advanced prostate cancer or spontaneous prostate cancer remission. In the context of cancer regression, reduction in TLO size correlated with massive decline in PSCA^+^ cells, enrichment in components associated with favorable tumor immunity (CD8 T cell accumulation, increase in HEV size), and the considerable reversion of local immunosuppression (notable reduction in COX2 cell density and Treg infiltration) and tumor neovascularization. By contrast, despite that PSCA was highly expressed by epithelial cells in advanced carcinoma tumors, the immunosuppressive and protumorigenic environment (COX2, CD105 neovessels, Treg) was likely overwhelming protective immunity generated in TLO. Nevertheless, the presence of lymphatics and HEV in TLO, even at advanced stages of prostate cancer, is indicating that dendritic cell or CD8 T cell-based therapies could be attractive adjuvant therapies, which can exploit the navigation systems of residual TLO to stimulate prostate cancer regression. It is also important to consider that the potential use of dendritic cells loaded with prostate antigens or antigen-specific T cells poses the risk of causing prostate damage. Nonetheless, the feasibility of using these cell therapies in humans is supported by preclinical studies of prophylactic vaccination with prostate antigens, which induced protective tumor immunity without development of detectable autoimmune disease (83, 84).

### Are Treg and COX2^+^ Inadvertently Impairing Tumor Immunity?

It is well known that Treg are critical in modulating excessive inflammation with the purpose of preventing damage to internal organs and thus preserving their physiological functions (50). For many years, it has been proposed that persistent inflammation leads to development of cancer (80–82). Thus, despite its infamous suppressive role, it is possible that Treg and COX2^+^ cells are simply trying to modulate chronic inflammation in the prostate to prevent progression toward malignancy. However, they are inadvertently interfering with the induction of tumor antigen-driven immune responses in TLO. Thus, a delicate balance between effector lymphocytes and Treg must exist in tumors to modulate inflammation without affecting protective tumor immunity. Although local increase in Treg seems to occur with the purpose of preventing local damage in micro-domains containing TLO, the tradeoff price is the progressive impairment of local tumor immunity, which compromises the life of prostate cancer patients.

### Prostate Has a Supportive Environment for Type 1 Immunity

Intriguingly, we confirmed that prostate tumors have permissive environments for the induction of type 1 immunity. Especially we detected considerable numbers of immune and epithelial cells that were producing significant amounts of CXCL10—a chemokine with potent angiostatic properties, which is also critical to attract CXCR3^+^ T cells and NK cells (60). One might expect that production of CXCL10 by epithelial cells will recruit T cell and NK cells to areas of epithelial cell transformation and accelerate tumor clearance. However, it has been reported that CXCR3A over expression on prostate tumor cells leads to invasion and metastasis (85). Thus, it is possible that during cancer progression production of CXCL10 by transformed epithelial cells might stimulate tumor metastasis. It is also known that COX2 is produced, in response to IFNγ and TNFα stimulation, with the purpose of modulating type 1 responses (49). According to the strong signal for CXCL10 in transformed epithelial cells, it is possible that attraction of CXCR3^+^ Th1 cells by transformed epithelial cells may enhance IFN-dependent production of COX2 in tumor areas, enhancing local tumor angiogenesis and immunosuppression. In addition, recent reports have shown that COX2-dependent production of PGE_2_ is responsible for stimulating PD-L1 expression on myeloid suppressor cells (86). Consistent with this idea, we found a considerable increase in the accumulation of PD-L1^+^ cells in tumor areas of patients at intermediate and advanced stages of prostate cancer. Thus, it seems PGE_2_ produced by epithelial cells might induce strong PD-L1-mediated immunosuppression in tumor areas during prostate cancer progression. Considering that current therapies for lung cancer are based in the blockade of PD-L1 to reactivate tumor immunity, we are also speculating that due the continuous PGE_2-_dependent induction of PD-L1 expression on myeloid suppressor cells, a synergistic therapy focused on blocking Treg and COX2 might be a more powerful approach to reverse immunosuppression in prostate cancer.

One of the limitations of our study was the logistical difficulty for following up of a larger cohort of prostate cancer patients. However, it was clear that although collection of small biopsies was sufficient to establish prostate cancer diagnosis and prognosis, large prostatectomy specimens were instrumental for performing a detailed examination of the molecular and cellular changes in different microenvironments of the malignant prostate. We propose that a detailed characterization of TLO in prostate tumors might be useful in the near future to stratify patients for personalized therapies based on TLO-associated cellular and molecular signatures. Also, it is possible that immune contexture of TLO can be used as a biomarker of response to classical therapies such as radiation, chemotherapy, and even anti-angiogenic therapies. The expectation is that release of tumor antigens by dying tumor cells will potentially enhance TLO formation and organization, indicating that therapies are making transformed epithelial cells visible for the immune system.

It is important to test the relevance of COX2 and Treg in preclinical models before moving to the clinical arena. However, we analyzed prostate from transgenic mouse model for prostate cancer (TRAMP) mice at various stages of spontaneous cancer development, but we did not find TLO (data not shown). A future alternative to induce TLO in TRAMP mice might rely on increasing local LIGHT expression to attract immune cells to prostate tumors (87, 88). Thus, the absence of TLO in animal models of prostate cancer complicates the design of experimental approaches to mechanistically confirm that COX2 and Treg are modulating TLO-driven tumor immunity. Nevertheless, we hope that our study can propel the interest for performing more retrospective studies with larger numbers of prostatectomy specimens, in order to confirm not only the association between TLO organization and tumor immunity but also to motivate the design of prophylactic or therapeutic interventions that take advantage of the already formed TLO to prevent progression or induce regression in tumors from prostate cancer patients.

In conclusion, our study is showing the dynamic and complex interplay that exists among three different processes intertwined during cancer progression: (1) induction of TLO by chronic inflammation, (2) inflammation-induced cell transformation, and (3) establishment of a potent immunosuppressive environment that impairs organization and protective functions of tumor-associated TLO in prostate cancer. Importantly, we detected a considerable increase in COX2 and Treg, two well-known players in the modulation of type 1 immunity, HEV integrity, and CD8 T cell accumulation, which are likely impacting tumor-associated TLO in prostate cancer. Although it is likely that Treg- and COX2-producing cells are perhaps trying to prevent inflammation-driven cell transformation and prostate damage, we propose that they might also impair local tumor immunity. Nevertheless, the risk-benefit of therapies in prostate cancer patients should be carefully assessed to decide whether induction of tissue damage is better than losing the invaluable opportunity for timely delivery of cell-based and/or COX2 blocking therapies to reverse tumor immune suppression, enhance tumor immunity in TLO, and prolong life expectancy of prostate cancer patients.

## Ethics Statement

Prostate specimens were collected with written consent of patients and after approval by the Ethical Committee of the National Institute of Medical Sciences and Nutrition “Salvador Zubiran.” All subjects gave written informed consent in accordance with the Declaration of Helsinki.

## Author Contributions

MG-H and JR-M: design, execution, analysis, drafting, and revision of manuscript; NU-U and RE-G provided human samples and clinical and pathological data. WK provided histological samples from TRAMP mice. All authors participated in interpretation of results, drafting, and critically reviewing the manuscript and approved the final version of the manuscript.

## Conflict of Interest Statement

The authors declare that the research was conducted in the absence of any commercial or financial relationships that could be construed as a potential conflict of interest.

## Supplementary Material

Figure S1 **Labeling of proliferating immune cells by antibodies specific for Ki-67 and proliferating cell nuclear antigen (PCNA)**. Consecutive serial sections of the same tonsil were stained with antibodies against **(A)** PCNA, CD8, and CD20 or **(B)** Ki-67, CD8, and CD20. Representative 200× pictures of triple immunofluorescent stain for Ki-67, PCNA, and CD20 are shown in **(C)** prostatic intraepithelial neoplasia (PIN), **(D)** intermediate, **(E)** advanced, and **(F)** evanescent prostate carcinoma. Germinal centers in tonsils and in a prostatectomy from intermediate prostate cancer patients are outlined with white dashed lines. Yellow arrows point to proliferating CD20^+^ B cells. Scale bar represents 100 μm.

Figure S2 **Measurement of areas covered by cells expressing prostate stem cell antigen (PSCA) (A) and CXCL10 (B) in prostatectomies**. To calculate the areas covered by PSCA^+^ and CXCL10^+^ cells in panoramic tumor areas, 3 × 3 mosaic pictures were taken with the Zeiss Axioplan microscope (1.043 mm^2^). Areas covered by PSCA and CXCL10 in JPGE panoramic pictures were blindly measured with NIH ImageJ software. Bar represent mean ± SEM. Statistically significant differences: ***p* ≤ 0.005, ****p* ≤ 0.0005.
