# Supplementary figures and images for: A Unique Cellular and Molecular Microenvironment Is Present in Tertiary Lymphoid Organs of Patients with Spontaneous Prostate Cancer Regression
Source: Front Immunol. 2017 May 17;8:563. doi: 10.3389/fimmu.2017.00563 (PMC5434117; doi:10.3389/fimmu.2017.00563)

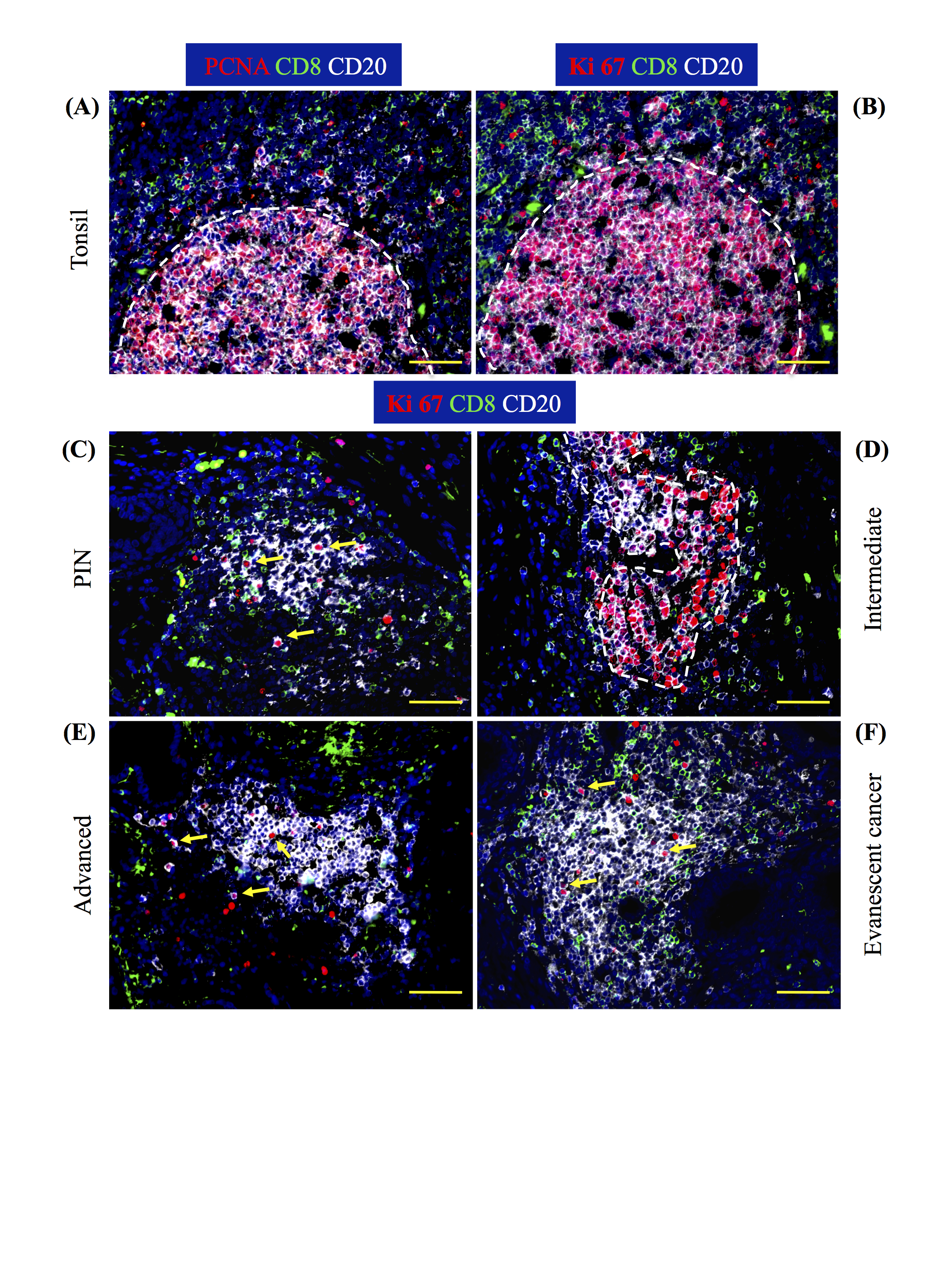

Supplement: Figure S1 — Labeling of proliferating immune cells by antibodies specific for Ki-67 and proliferating cell nuclear antigen (PCNA). Consecutive serial sections of the same tonsil were stained with antibodies against (A) PCNA, CD8, and CD20 or (B) Ki-67, CD8, and CD20. Representative 200× pictures of triple immunofluorescent stain for Ki-67, PCNA, and CD20 are shown in (C) prostatic intraepithelial neoplasia (PIN), (D) intermediate, (E) advanced, and (F) evanescent prostate carcinoma. Germinal centers in tonsils and in a prostatectomy from intermediate prostate cancer patients are outlined with white dashed lines. Yellow arrows point to proliferating CD20+ B cells. Scale bar represents 100 μm. [file Image_1.JPEG]

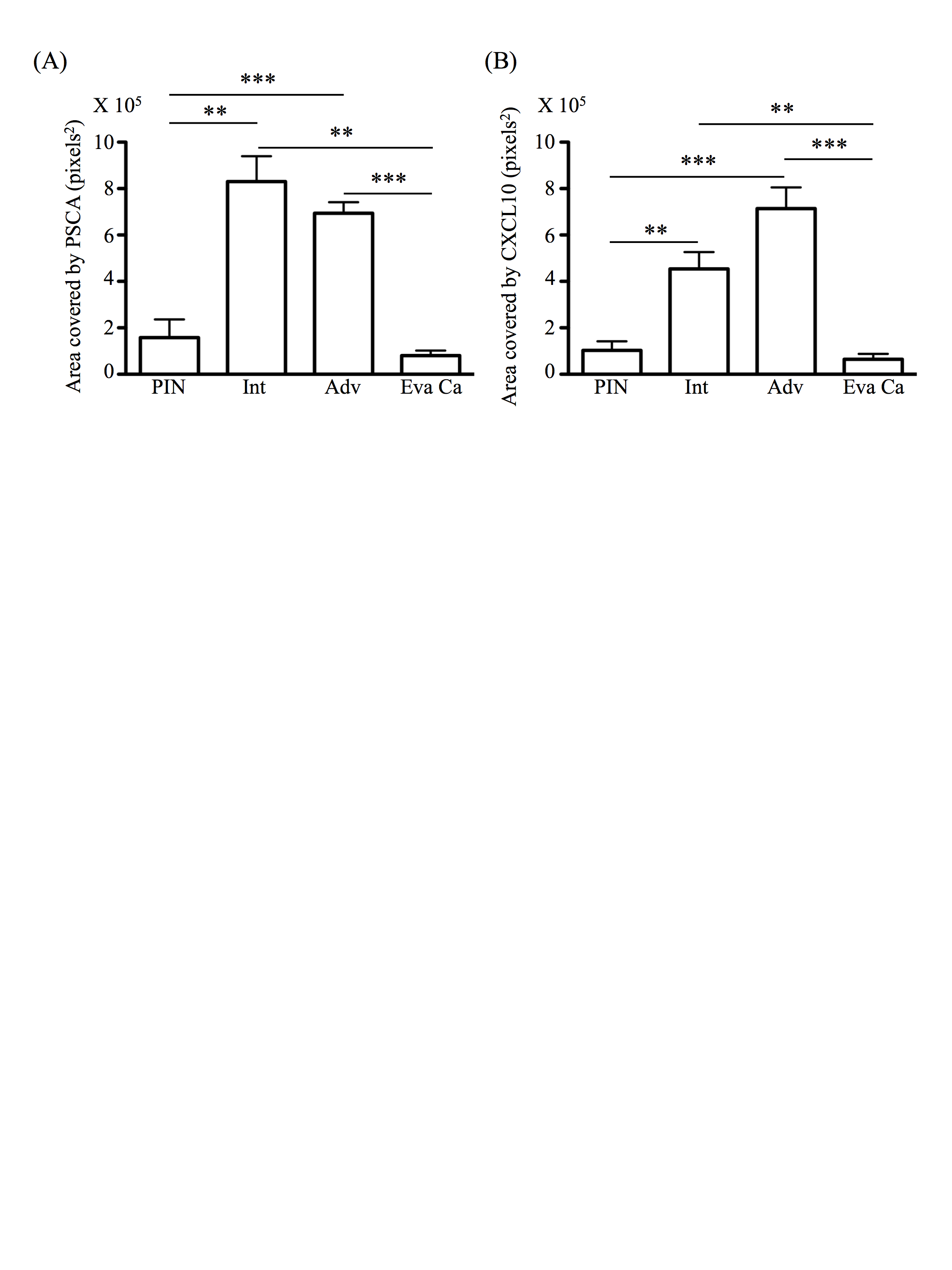

Supplement: Figure S2 — Measurement of areas covered by cells expressing prostate stem cell antigen (PSCA) (A) and CXCL10 (B) in prostatectomies. To calculate the areas covered by PSCA+ and CXCL10+ cells in panoramic tumor areas, 3 × 3 mosaic pictures were taken with the Zeiss Axioplan microscope (1.043 mm2). Areas covered by PSCA and CXCL10 in JPGE panoramic pictures were blindly measured with NIH ImageJ software. Bar represent mean ± SEM. Statistically significant differences: **p ≤ 0.005, ***p ≤ 0.0005. [file Image_2.JPEG]
